# Supplementary material for: Regulatory roles of three-dimensional structures of chromatin domains
Source: Genome Biol. 2025 Jun 27;26:184. doi: 10.1186/s13059-025-03659-7 (PMC12203726; doi:10.1186/s13059-025-03659-7)
Supplement: Supplementary file 1 — Additional file 1: Supplementary Figures S1–S38. [file 13059_2025_3659_MOESM1_ESM.pdf]

Supplementary figures

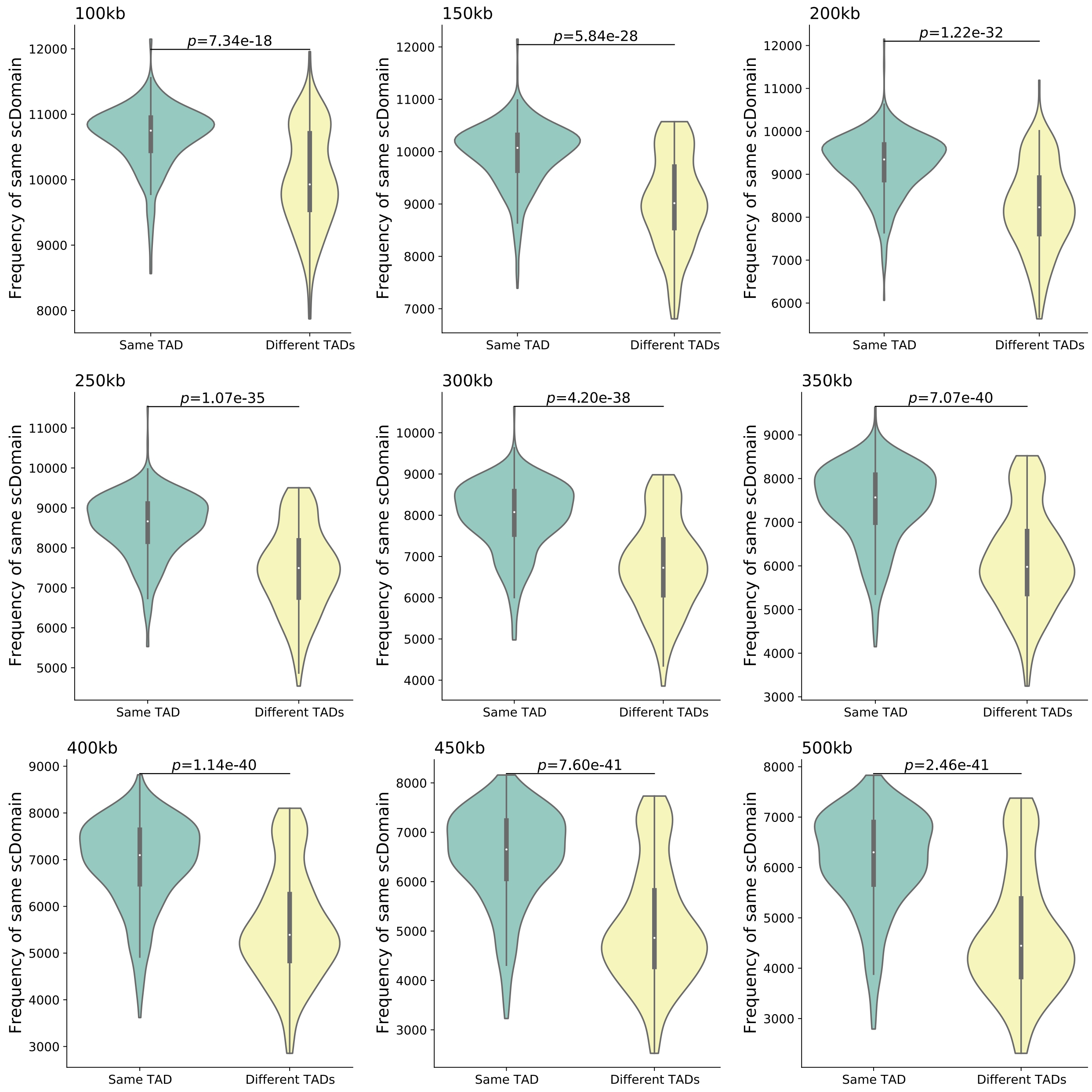

Figure S1: Consistency between scDomains and TADs of IMR90 cells. Each panel includes pairs of 50kb regions separated by a certain genomic distance (100kb-500kb). Each region pair is classified as either belonging to the same TAD or not. The frequency (i.e., number of cells among a total of ∼12,000 cells) that the two regions belonging to the same scDomain is then counted. Finally, these frequency values from all region pairs are collected and visualized using violin plots. p-values are computed using the two-sided Mann–Whitney U test.

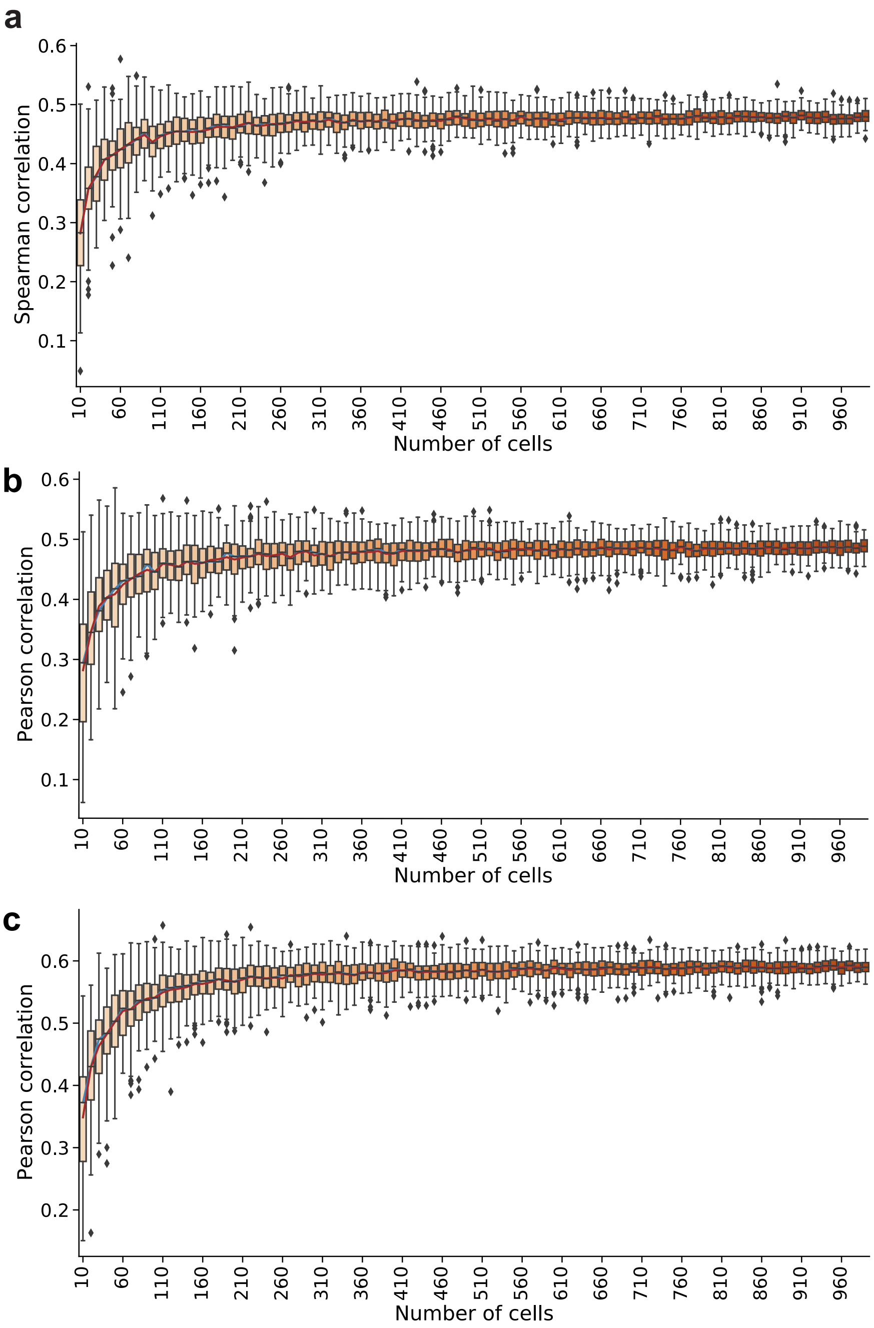

Figure S2: Consistency between average intra-scDomain ratios and intra-TAD ratios of IMR90 cells. (a-c) Distribution of correlations between intra-TAD ratio and average intra-scDomain ratio in x single cells, for x equal to 10-990, based on TADs called by directionality index (a), insulation score (b), or TopDom (c). For each value of x, we first sub-sampled x single cells and computed the average intra-scDomain ratio of each 50kb region over these cells. Then the vector of these average intra-scDomain ratios and the corresponding vector of intra-TAD ratios of the same 50kb regions were taken to compute a correlation. Finally, by repeating the sub-sampling of x cells 100 times, we obtained a distribution of correlation values.

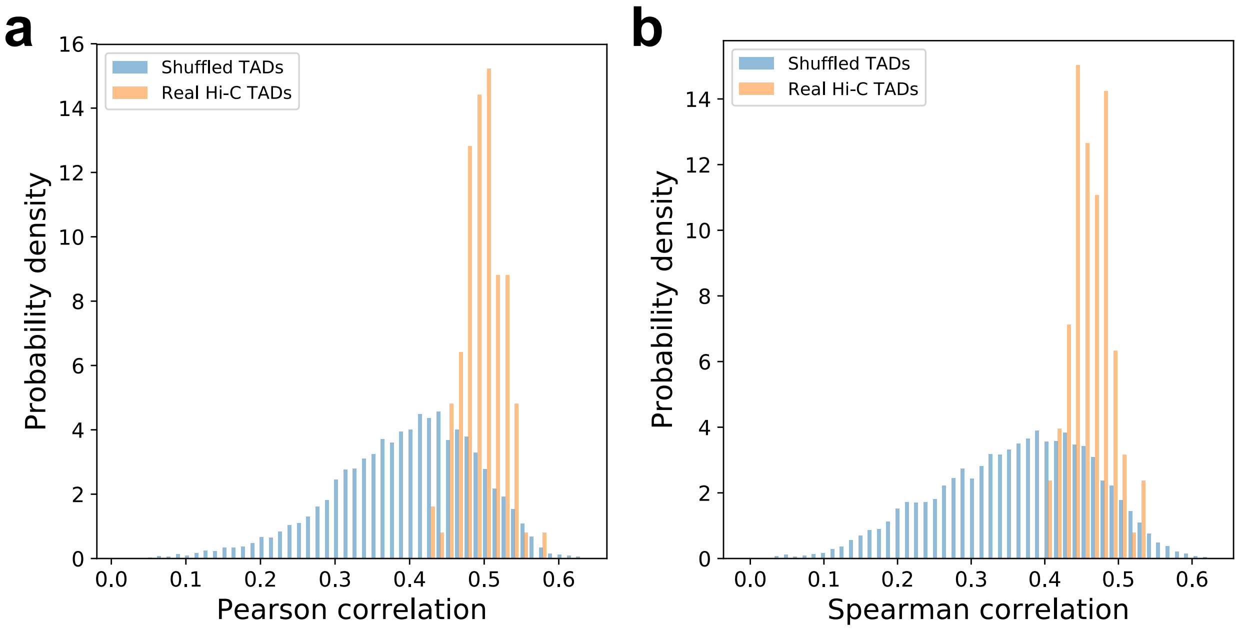

Figure S3: Comparison of correlations between intra-TAD ratio and intra-scDomain ratio for real and randomly shuffled TADs. Histogram of Pearson (b) and Spearman (c) correlations when considering $x = 200$ cells, based on real TADs or shuffled TADs.

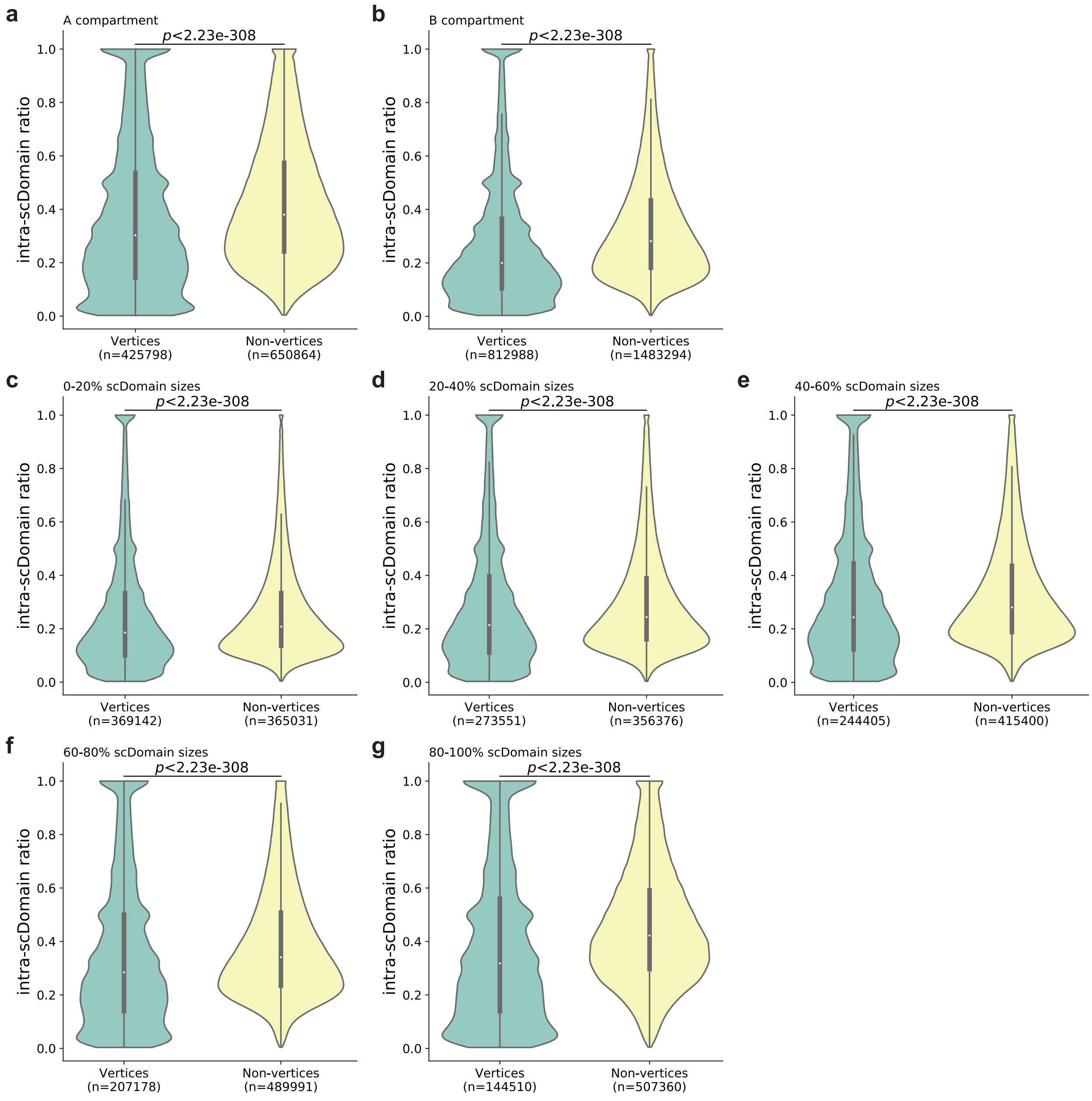

Figure S4: Comparing the intra-scDomain ratios of regions on the convex hulls of scDomains (vertices) and other regions (non-vertices). In each panel, a violin plot is shown for all included vertices from all imaged single cells, and a separate violin plot is shown for all included non-vertices from all imaged single cells. Different subsets of regions were included in the different panels, namely only regions in the A (a) and B (b) compartments, and only regions within a certain size range according to the number of constituent 50kb regions, namely 0-20 percentiles (c), 20-40 percentiles (d), 40-60 percentiles (e), 60-80 percentiles (f), and 80-100 percentiles (g), where a smaller percentile corresponds to scDomains with fewer constituent regions. p-values in all panels are computed using the two-sided Mann–Whitney U test.

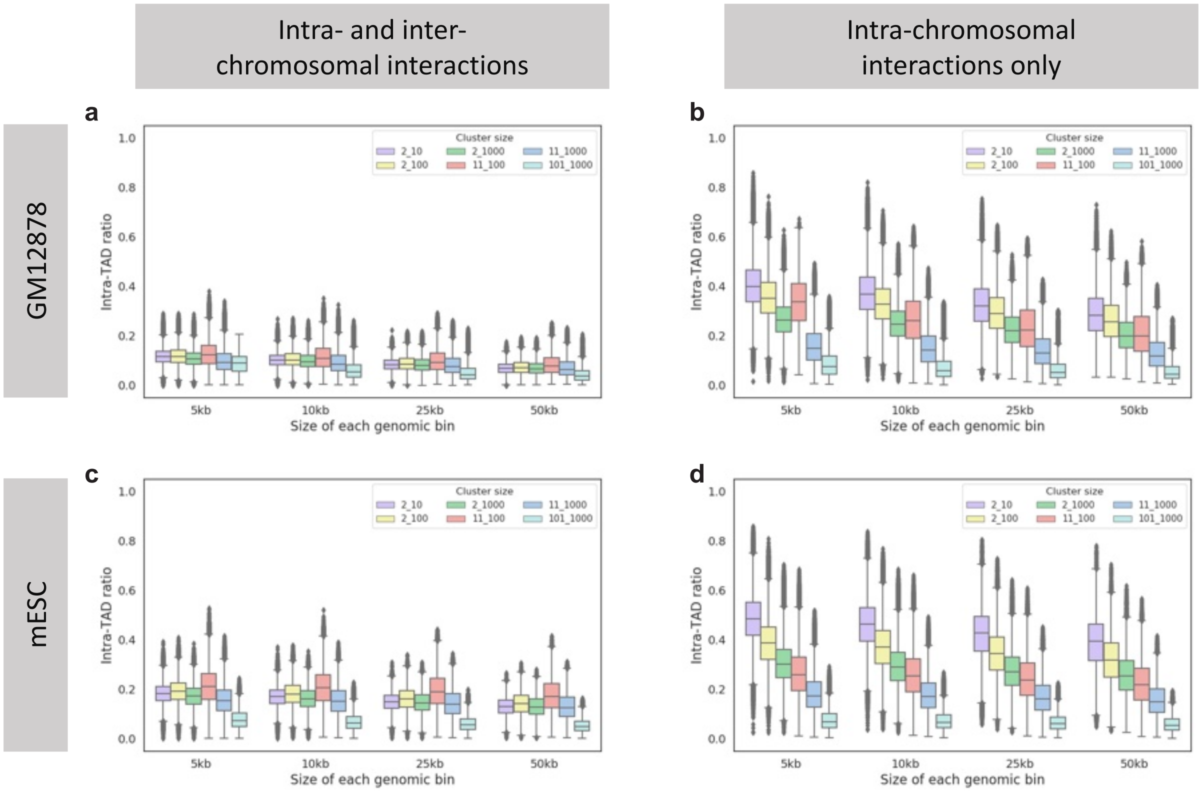

Figure S5: Effects of technical parameters on the intra-TAD ratio. The different panels show the intra-TAD ratios calculated using data from GM12878 cells (a,b) and mESCs (c,d), with both intra- and inter-chromosomal interactions (a,c) or only intra-chromosomal interactions (b,d). In each panel, the different groups show the intra-TAD ratios calculated with a specific size of genomic bins (5kb, 10kb, 25kb, or 50kb). Within a group, the different box plots correspond to different subsets of SPRITE clusters involved in the calculation of intra-TAD ratios selected based on their sizes, where x_y means the inclusion of only SPRITE clusters with at least x and at most y reads.

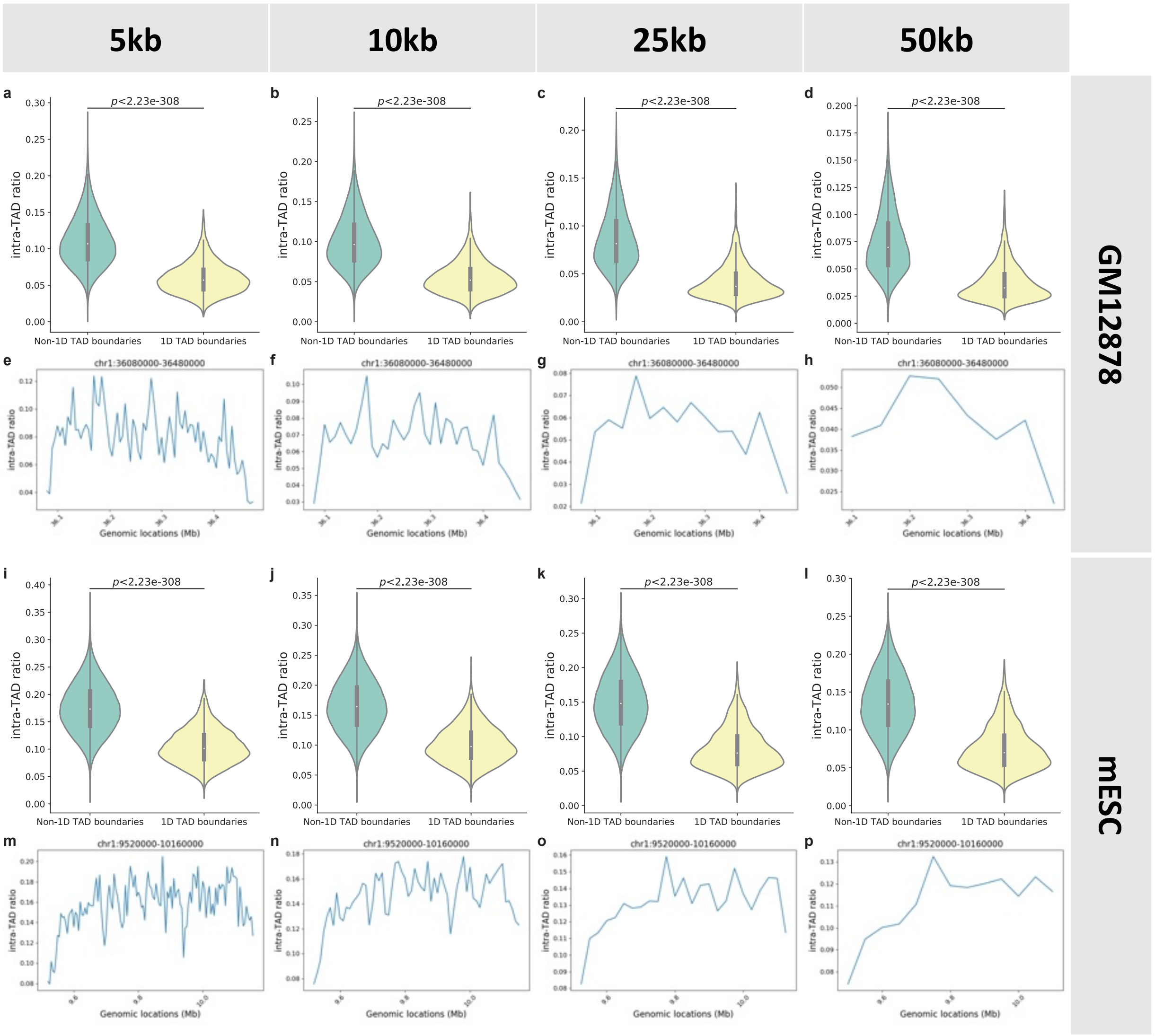

Figure S6: Comparing the intra-TAD ratios of 1D TAD boundaries and other TAD bins. The different panels show the intra-TAD ratios in GM12878 cells (a-h) and mESCs (i-p), at 5kb (a,e,i,m), 10kb (b,f,j,n), 25k (c,g,k,o), and 50kb (d,h,l,p) bin sizes. The violin plots (a-d, i-l) compare the distributions of intra-TAD ratios of genomic bins that overlap 1D TAD boundaries and those that do not. The line plots (e-h, m-p) show the change of intra-TAD ratio along the genomic span of example TADs.

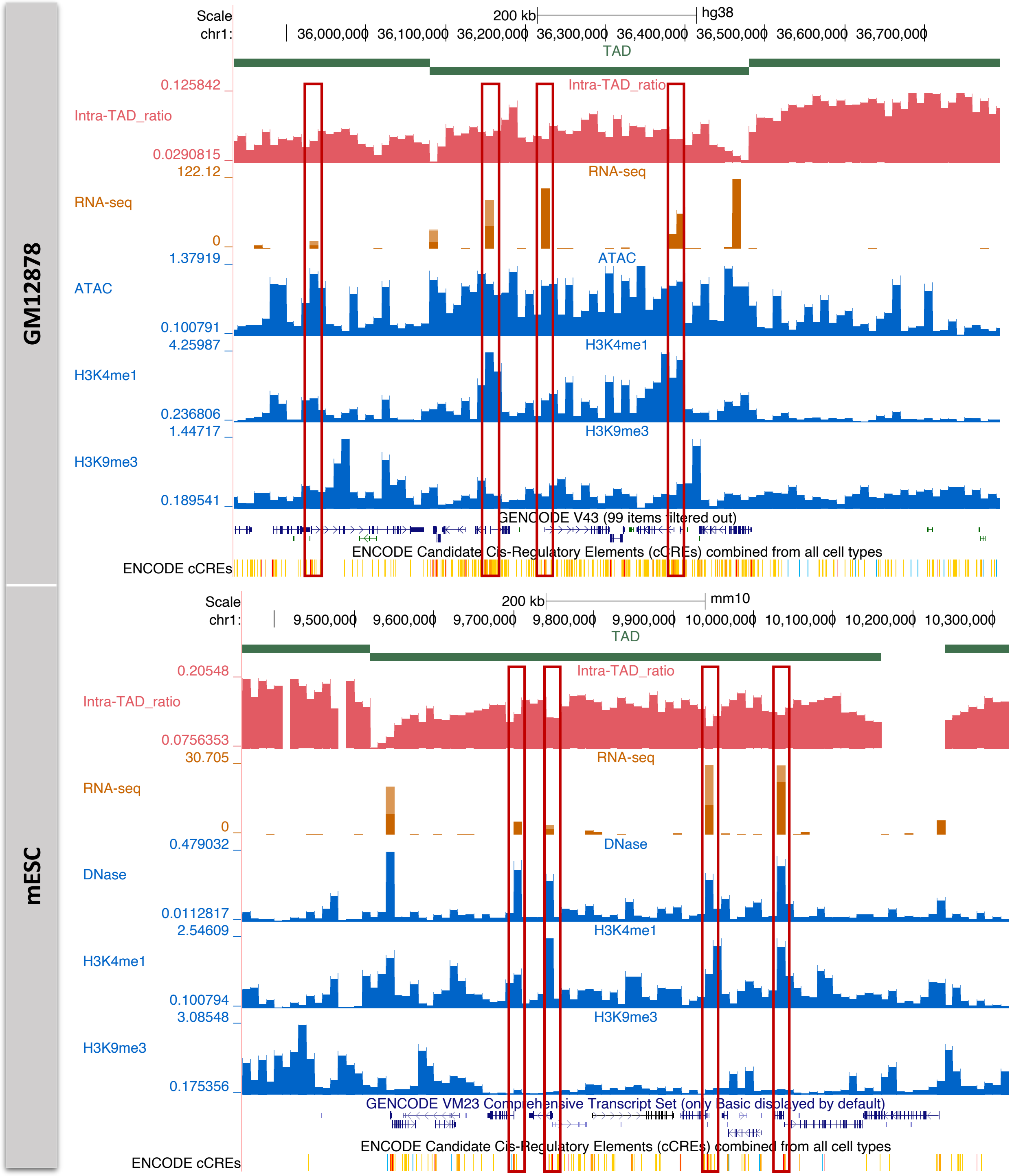

Figure S7: Genomic tracks showing TAD locations, intra-TAD ratios, RNA expression levels, signals of chromatin accessibility, H3K4me1, and H3K9me3, and gene annotation in GM12878 (a) and mESC (b). In each panel, the red boxes show example regions inside a TAD that are not close to 1D TAD boundaries but have a local dip of intra-TAD ratio, a corresponding high level of RNA expression, some enrichment of active chromatin signals and some depletion of inactive repressive chromatin signals.

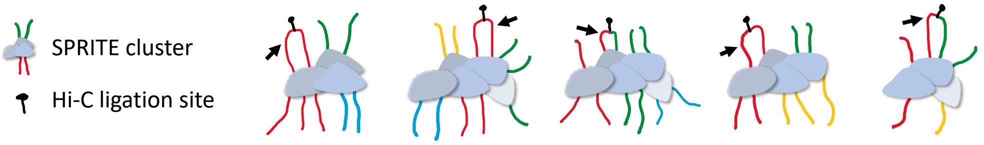

Bulk Hi-C		Read 1	Read 2	Read 3	Read 4	Read 5	Intra-TAD ratio
	Intra-TAD interactions	1	1	0	1	0	0.6
SPRITE		Cluster 1	Cluster 2	Cluster 3	Cluster 4	Cluster 5	Averaged intra-TAD ratio
	Intra-TAD interactions	4	3	3	3	2	/
	Total interactions	8	10	10	8	5	/
	Intra-TAD ratio	0.5	0.3	0.3	0.375	0.4	0.375

Figure S8: Calculations of intra-TAD ratio from SPRITE and bulk Hi-C data. Black arrows indicate the target DNA fragment of which the intra-TAD ratio is computed. DNA fragments from the same TAD are shown in the same color. Hi-C captures only one DNA fragment ligated to the target fragment at a time, while SPRITE captures the whole complex of multiple DNA fragments at the same time.

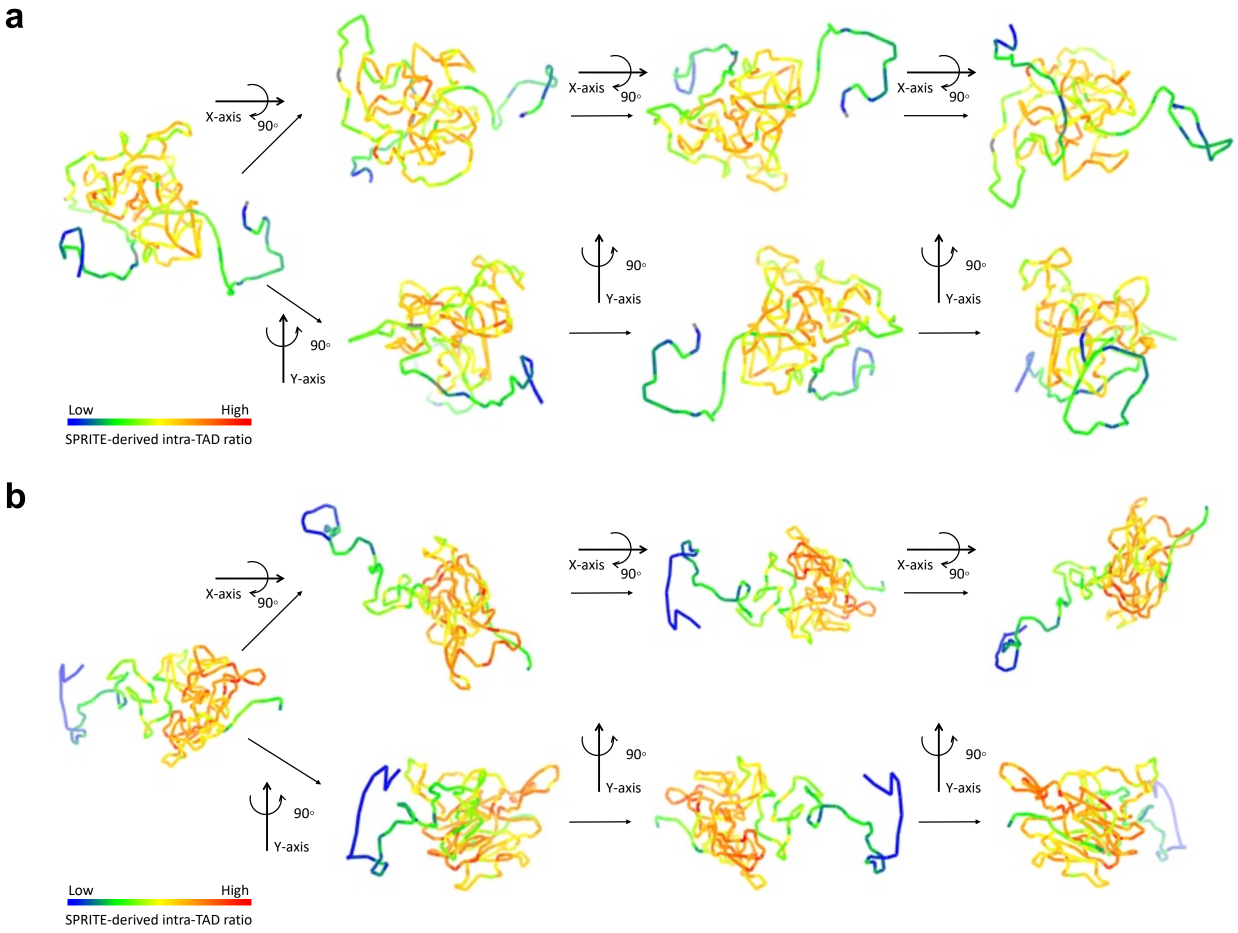

Figure S9: Visualization of intra-TAD ratios in structural models of two example TADs. The structural models of the first example TAD (chr18:77920000-80200000) (a) and the second example TAD (chr16:73000000-75560000) (b) were taken from Meng et al.. Each genomic bin is colored by its SPRITE-derived intra-TAD ratio. Gray indicates missing data.

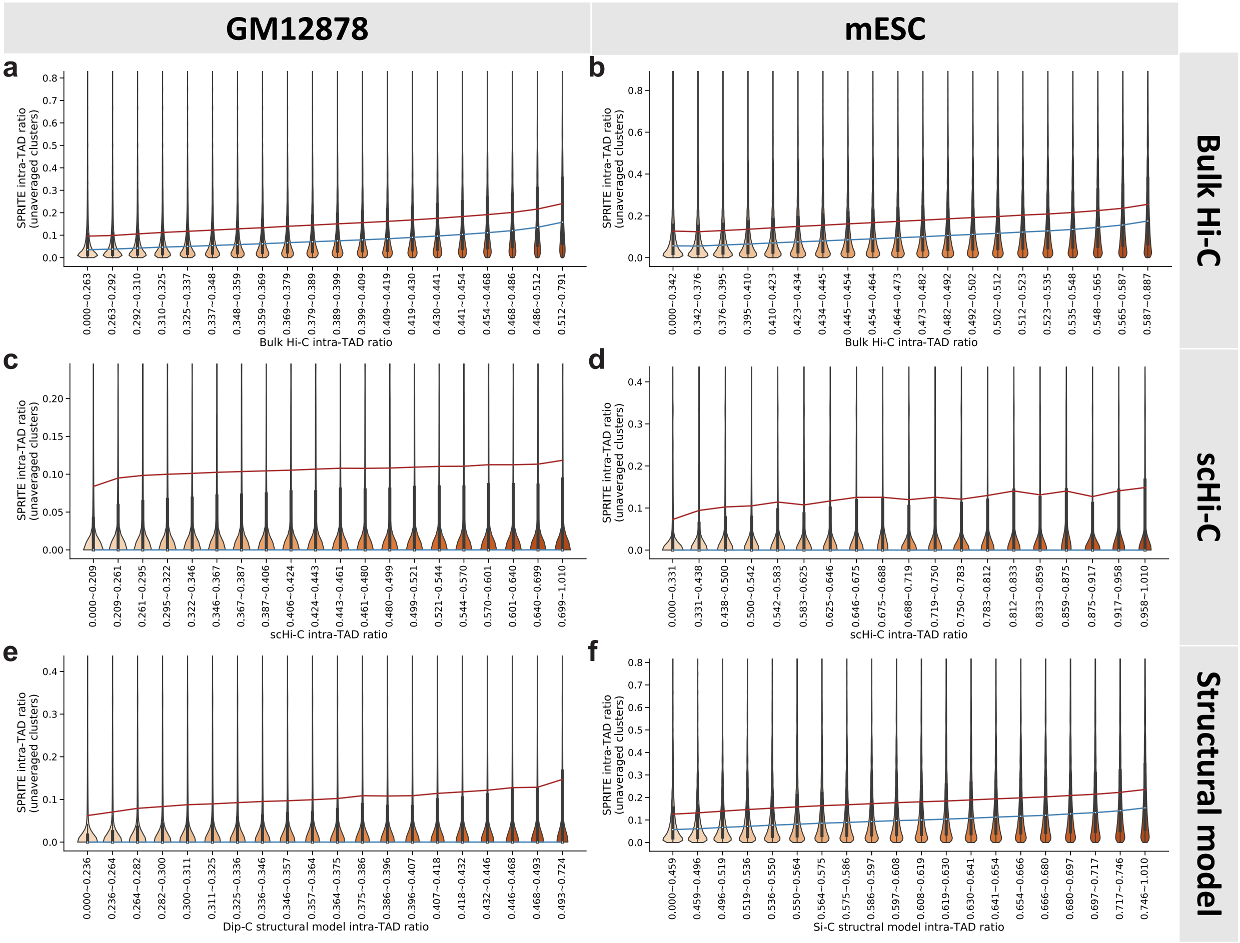

Figure S10: Comparing intra-TAD ratios calculated from SPRITE data without averaging over SPRITE clusters and intra-TAD ratios calculated from other types of data. Genomic bins were put into discrete classes based on their intra-TAD ratios computed from bulk Hi-C (a,b), scHi-C (c,d), or structural models (e,f). The intra-TAD ratios computed from individual SPRITE clusters of the bins belonging to each class were then visualized using a violin plot. In all panels, the blue and red lines connect median and mean values of the different groups, respectively. PCC: Pearson correlation. SCC: Spearman correlation.

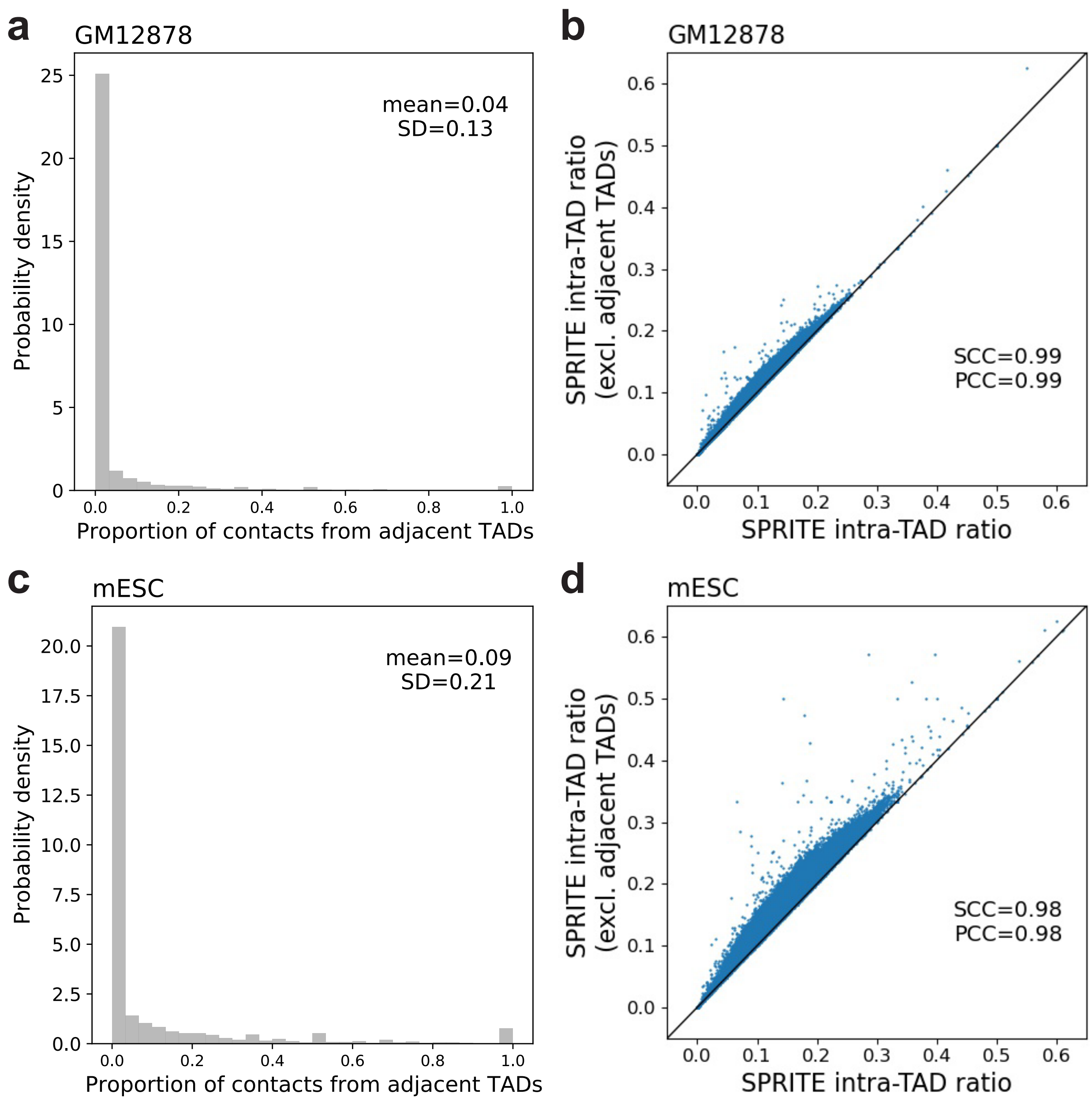

Figure S11: Effects of contacts from adjacent TADs in SPRITE data on the intra-TAD ratio. (a,c) Distribution of the proportion of contacts from adjacent TADs among all the inter-TAD contacts in GM12878 (a) and mESC (c). (b,d) Scatter plots of the intra-TAD ratio when interactions between adjacent TADs are excluded against the intra-TAD ratio when all the interactions are considered in GM12878 (b) and mESC (d).

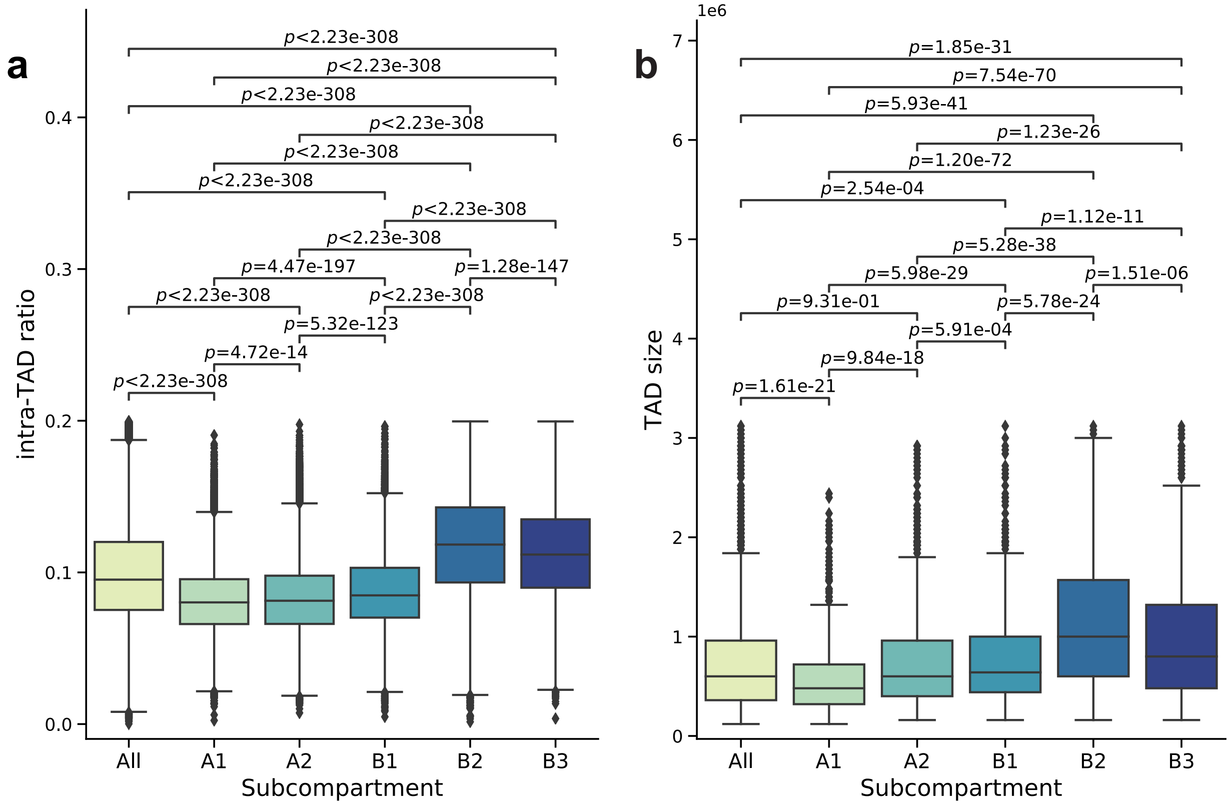

Figure S12: Distribution of intra-TAD ratio (a) and TAD size (b) of either all TADs or only TADs in a genomic subcompartment. The size of a TAD is defined as the length of its 1D genomic span.

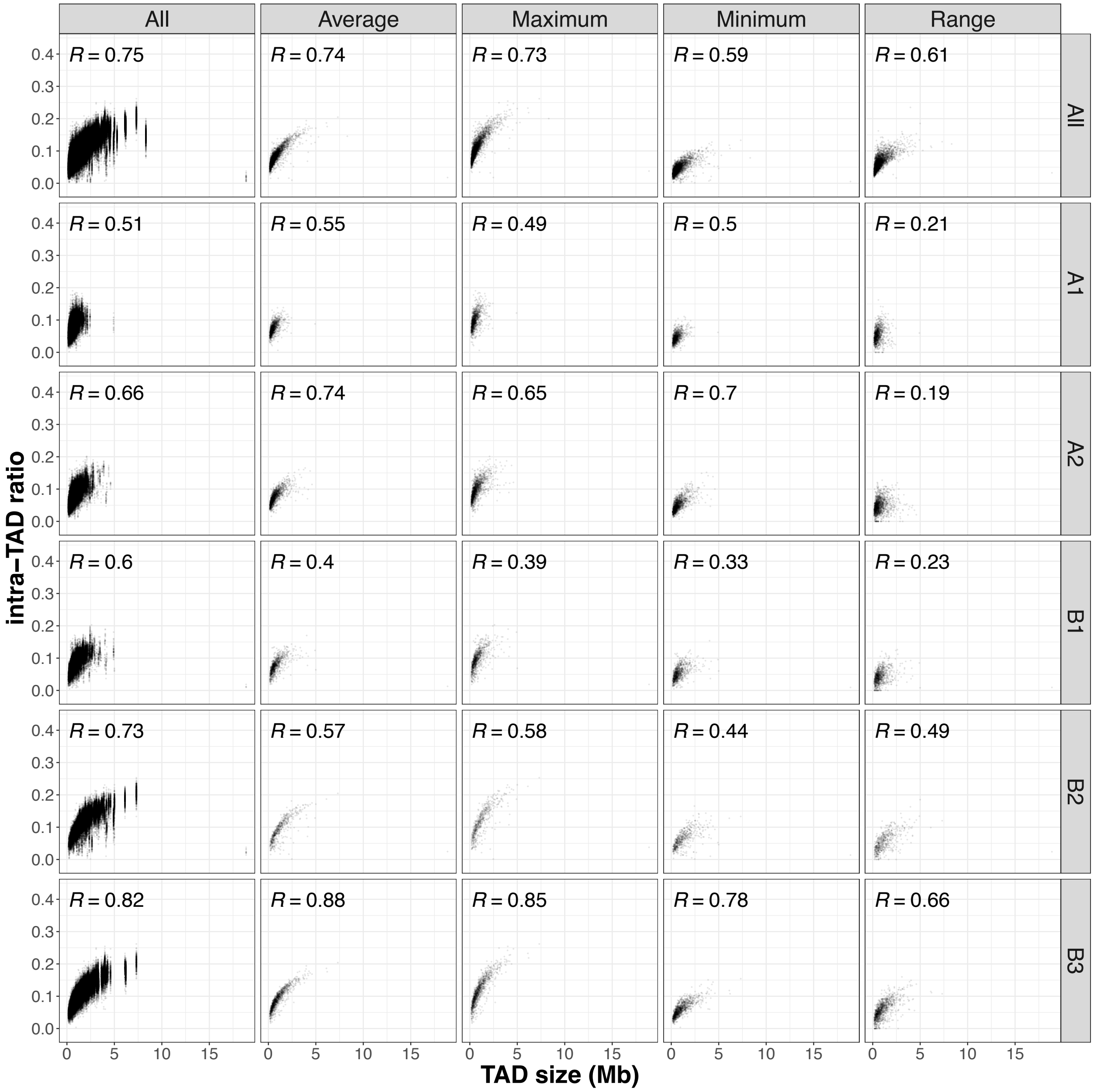

Figure S13: Relationship between intra-TAD ratio and TAD size. Each scatterplot compares TAD size with intra-TAD ratio based on either all TADs or only TADs in a genomic subcompartment (rows) and a specific way to aggregate intra-TAD ratios of all genomic bins in a TAD (columns).

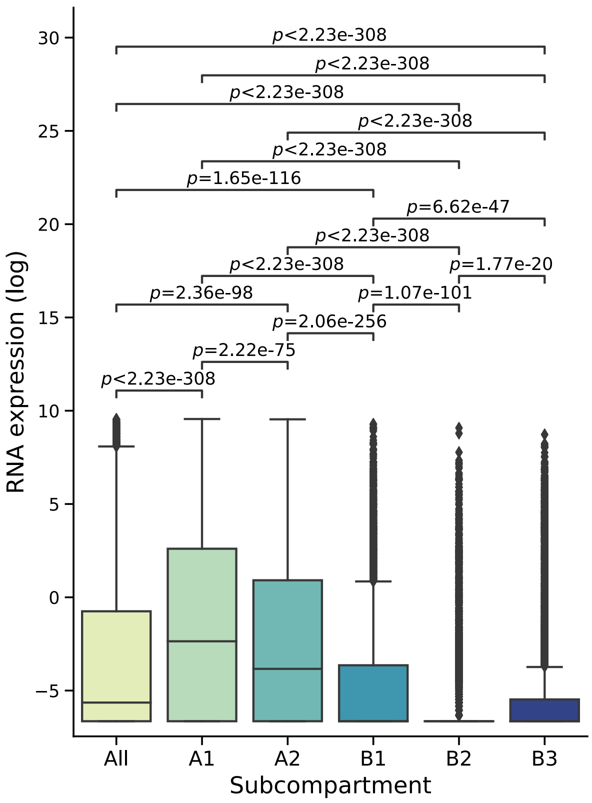

Figure S14: Distribution of expression levels of genes either in the whole genome or in a genomic subcompartment. RNA expression is quantified by $\log_2$(FPKM+0.01).

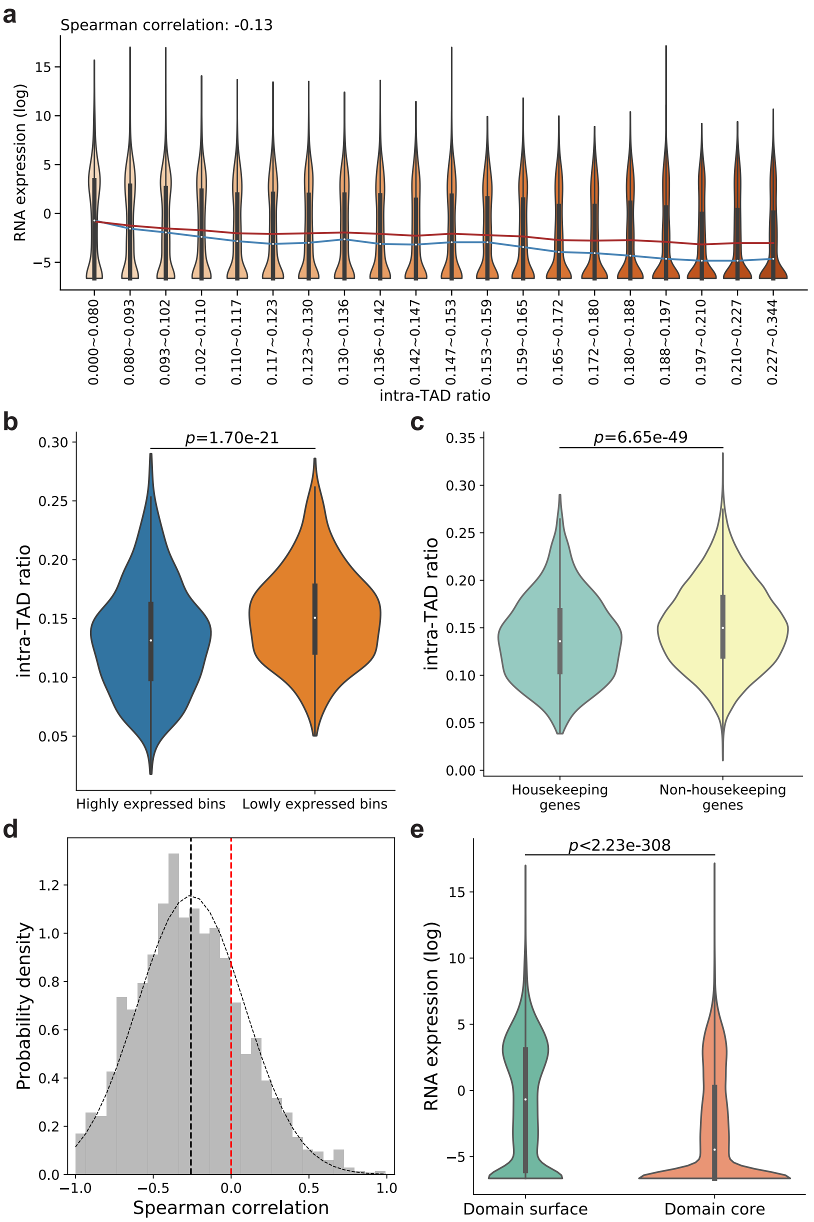

Figure S15: Inverse relationship between intra-TAD ratio and gene expression in mESCs. (a) Violin plots of gene expression ($\log_2$(FPKM+0.01)) of TSS bins in groups with increasing intra-TAD ratio and similar number of bins. The blue and red lines connect median and mean values of the different groups, respectively. (b) Violin plots of intra-TAD ratios of the highly expressed bins and lowly expressed bins, defined as the genomic bins containing TSSs of genes whose FPKM values rank within top and bottom 1,000, respectively. (c) Violin plots of intra-TAD ratio of housekeeing genes and non-housekeeping genes. (d) Distribution of Spearman correlations between gene expression and intra-TAD ratio of genomic bins in individual TADs. Black dotted curve shows the fitted normal distribution. The vertical black dotted line shows the mean of Spearman correlations. The vertical red dotted line shows zero Spearman correlation. (e) Violin plots of gene expression levels ($\log_2$(FPKM+0.01)) in domain surface bins and domain core bins. p-values in all panels are calculated using the two-sided Mann-Whitney U test.

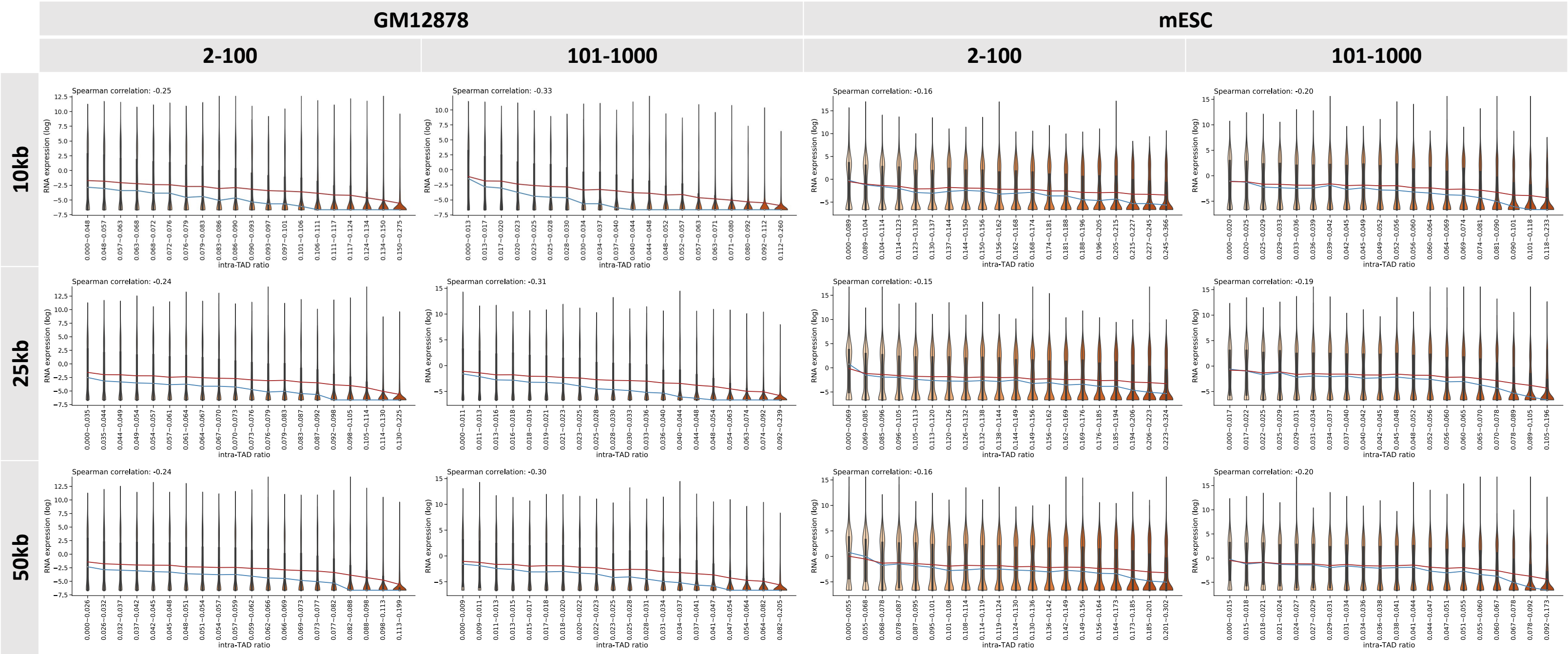

Figure S16: Inverse relationship between intra-TAD ratio and gene expression in GM12878 and mESC with different bin sizes (rows) and SPRITE cluster sizes (columns). Violin plots show gene expression ($\log_2$(FPKM+0.01)) of TSS bins in groups with increasing intra-TAD ratio and similar number of bins. The blue and red lines connect median and mean values of the different groups, respectively.

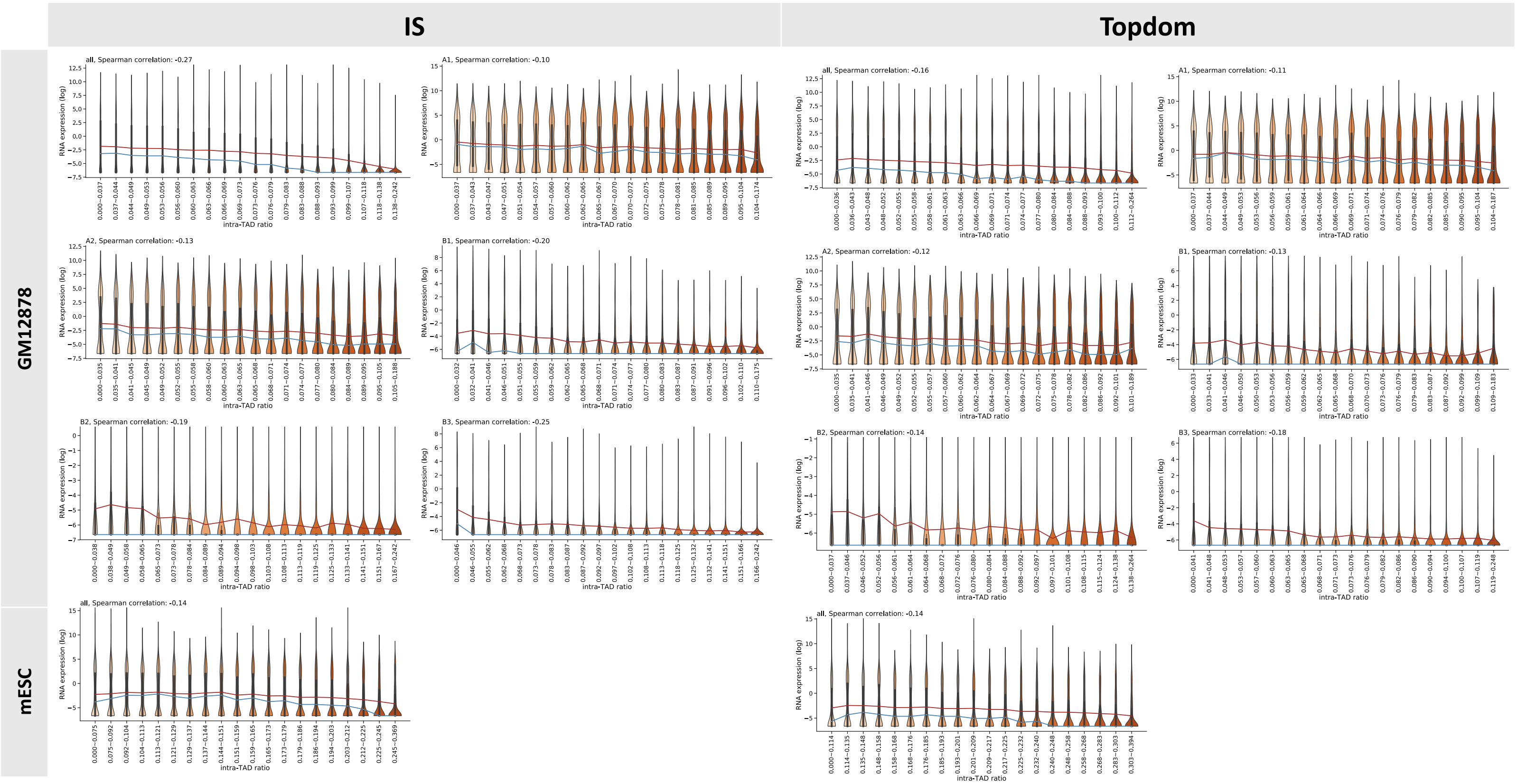

Figure S17: Relationship between intra-TAD ratio and gene expression GM12878 and mESC using insulation score (IS) and TopDom to call TADs. Violin plots show gene expression ($\log_2$(FPKM+0.01)) of TSS bins in groups with increasing intra-TAD ratio and similar number of bins. The blue and red lines connect median and mean values of the different groups, respectively.

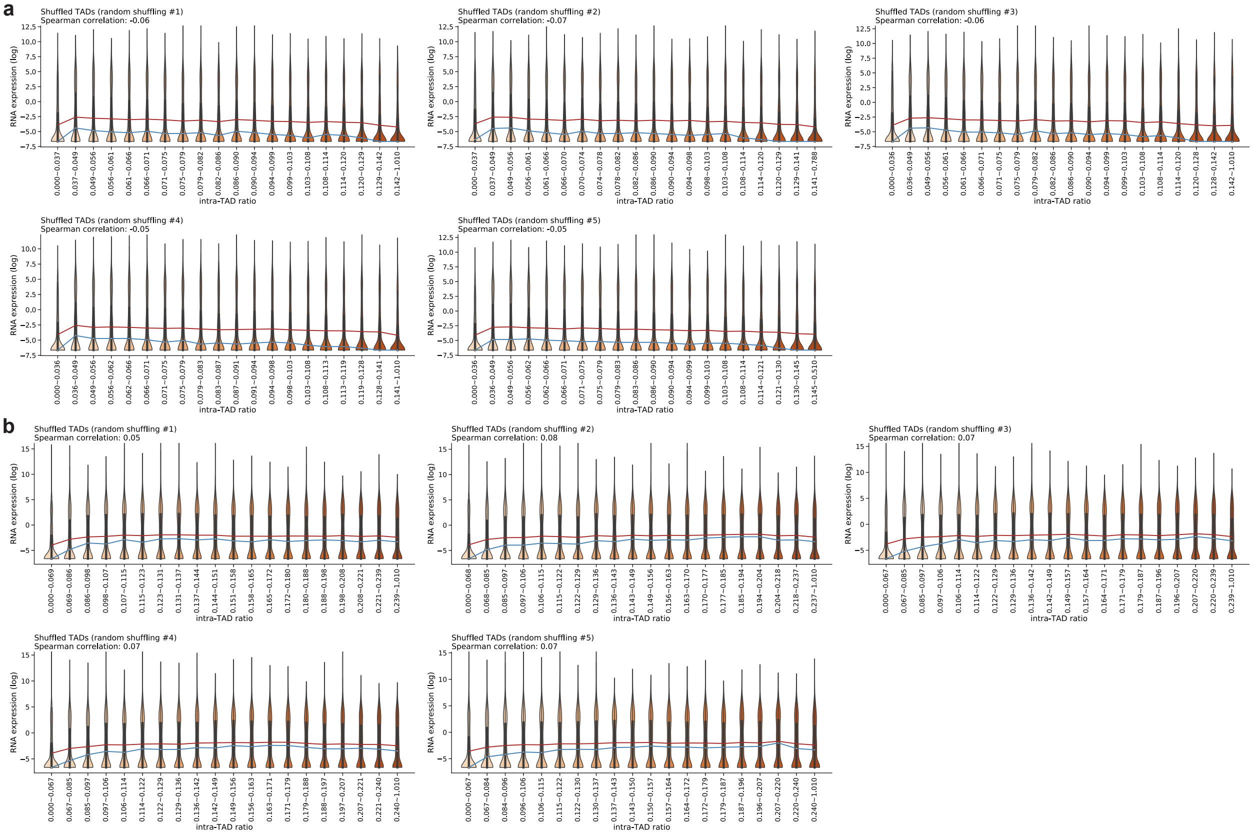

Figure S18: Relationship between intra-TAD ratio and gene expression for shuffled TADs in GM12878 (a) and mESC(b). Violin plots show gene expression ($\log_2$(FPKM+0.01)) of TSS bins in groups with increasing intra-TAD ratio and similar number of bins, with intra-TAD ratio being calculated using five sets of shuffled TADs. The blue and red lines connect median and mean values of the different groups, respectively.

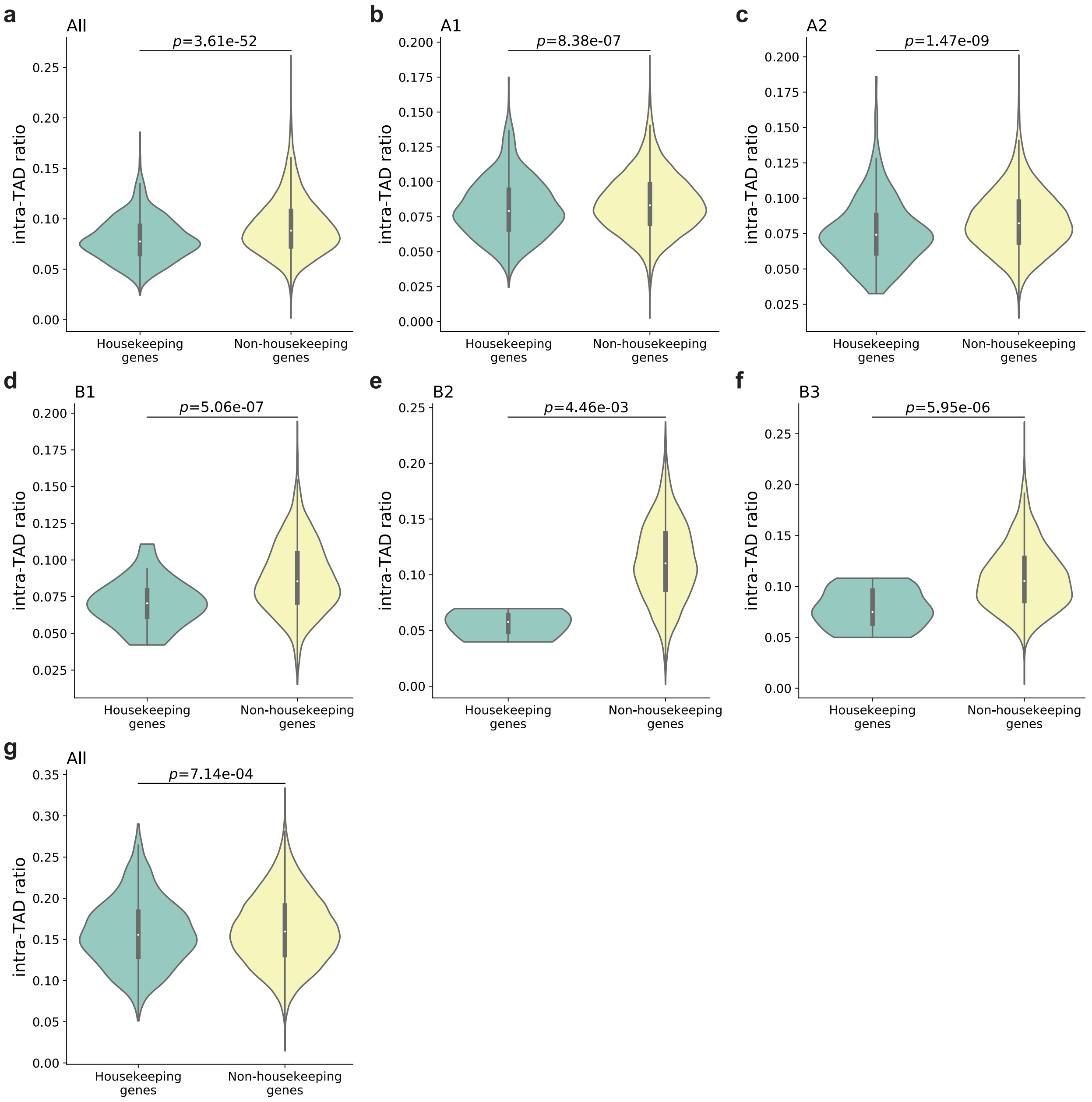

Figure S19: Violin plots of intra-TAD ratio of housekeeping genes and non-housekeeping genes when TAD boundaries and their neighboring regions were excluded in GM12878 (a-f) and mESC (g). p-values in all panels are calculated using the two-sided Mann-Whitney U test.

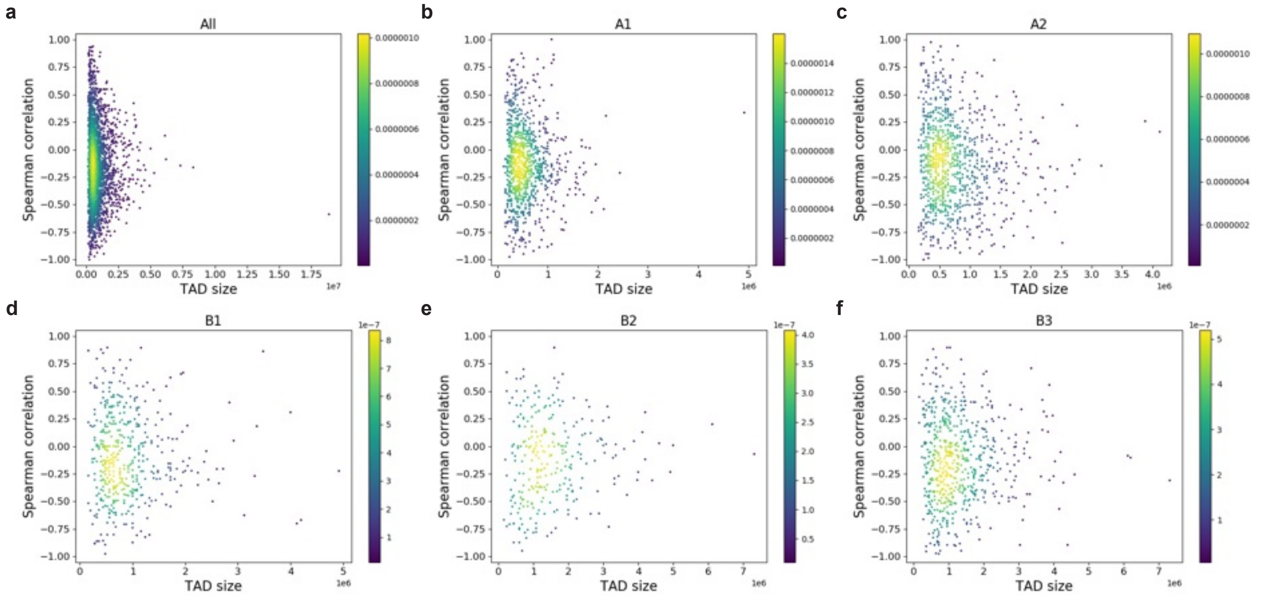

Figure S20: Density plots showing the Spearman correlation between intra-TAD ratio and gene expression of individual TADs versus TAD size for either all genomic genes (a) or genes in a genomic subcompartment (b-f).

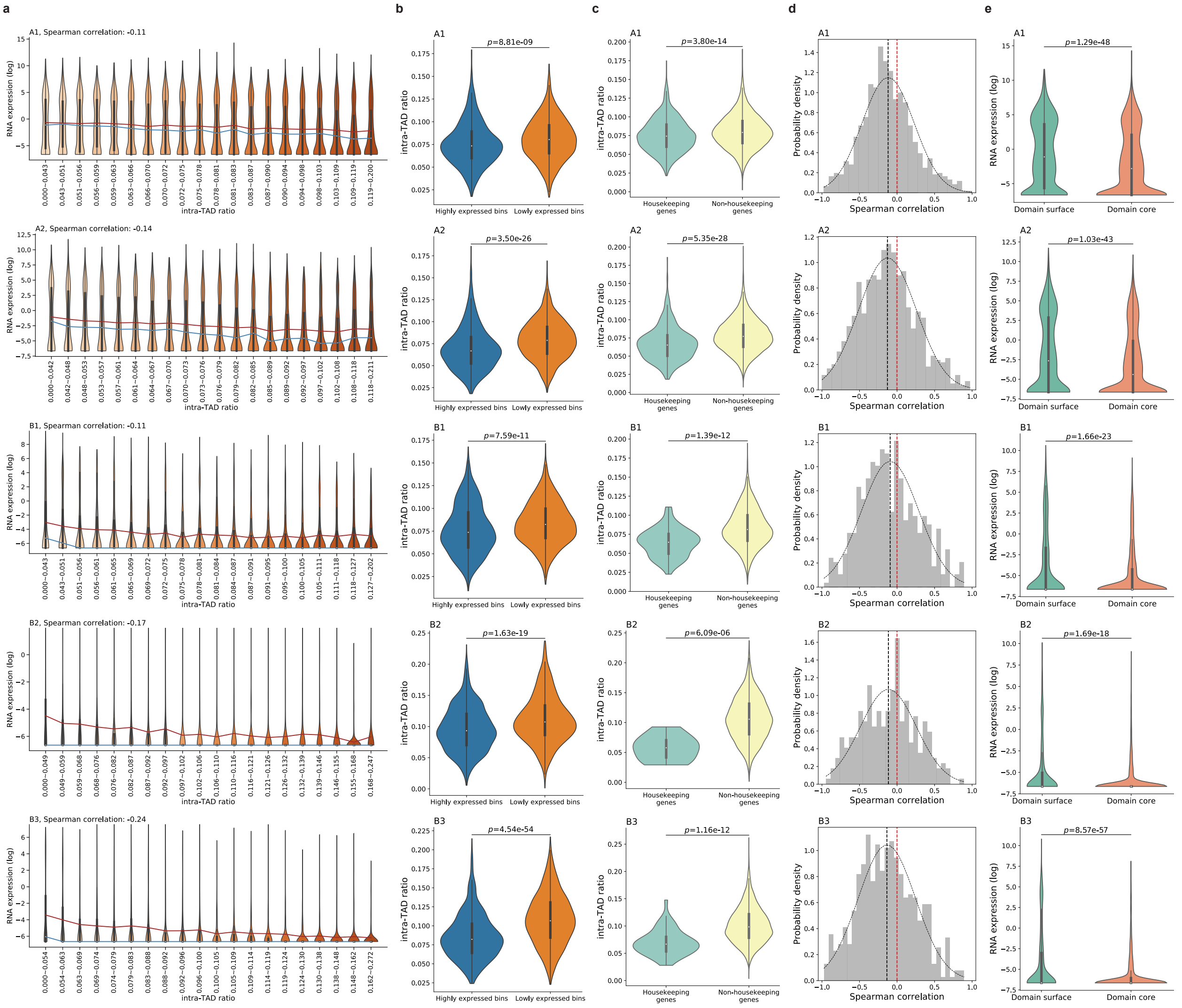

Figure S21: Intra-TAD ratio and gene expression have an inverse relationship in GM12878 for genes in individual subcompartments. (a) Violin plots of gene expression ($\log_2$(FPKM+0.01)) of TSS bins in groups with increasing intra-TAD ratio and similar number of bins. The blue and red lines connect median and mean values of the different groups, respectively. (b) Violin plots of intra-TAD ratios of the highly expressed bins and lowly expressed bins, defined as the genomic bins containing TSSs of genes whose FPKM values rank within top and bottom 1,000, respectively. (c) Violin plots of intra-TAD ratio of housekeeing genes and non-housekeeping genes. (d) Distribution of Spearman correlations between gene expression and intra-TAD ratio of genomic bins in individual TADs. Black dotted curve shows the fitted normal distribution. The vertical black dotted line shows the mean of Spearman correlations. The vertical red dotted line shows zero Spearman correlation. (e) Violin plots of gene expression levels ($\log_2$(FPKM+0.01)) in domain surface bins and domain core bins. p-values in all panels are calculated using the two-sided Mann-Whitney U test.

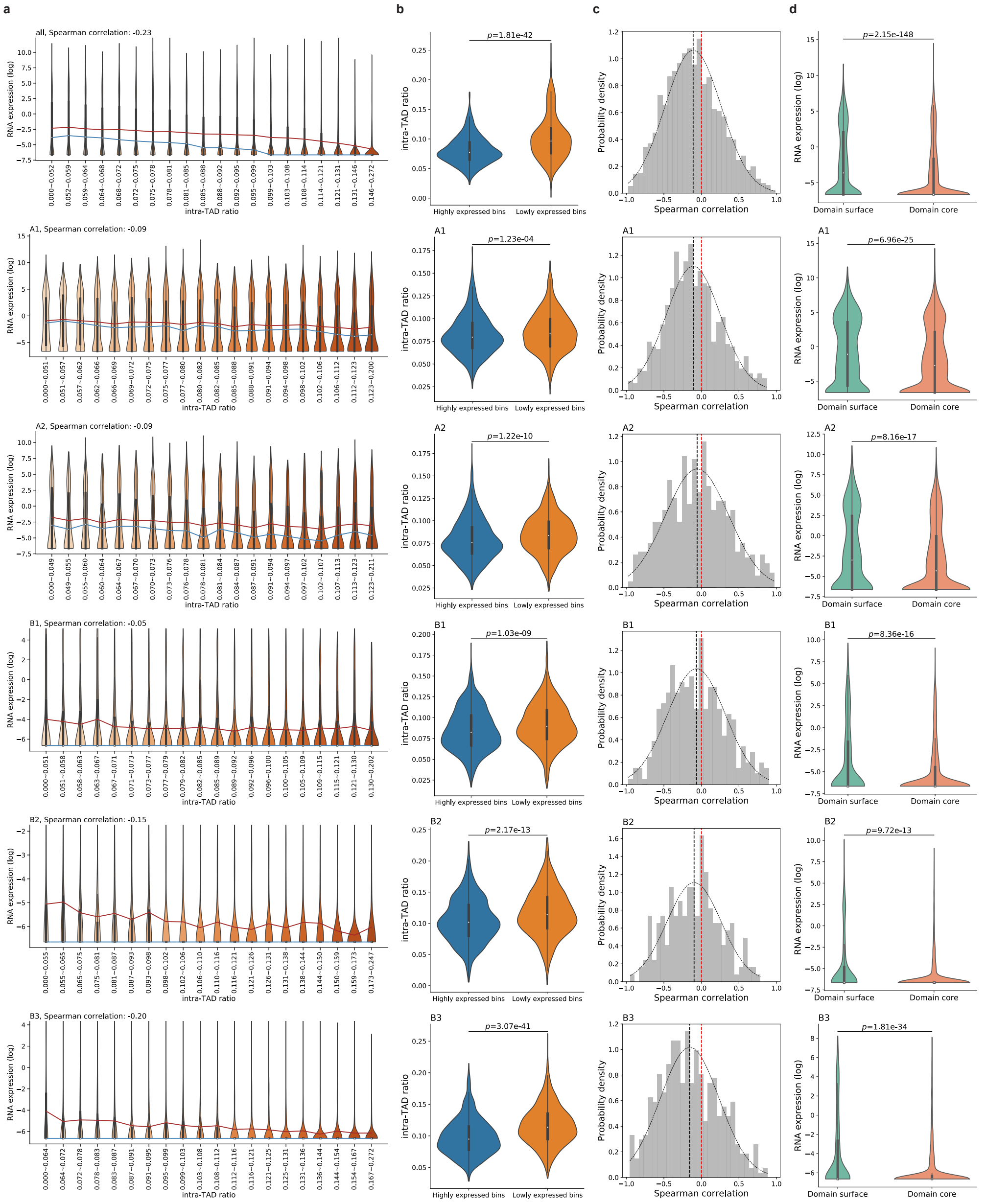

Figure S22: Intra-TAD ratio and gene expression have an inverse relationship in GM12878 when 1D TAD boundaries are excluded. (a) Violin plots of gene expression ($\log_2$(FPKM+0.01)) of TSS bins in groups with increasing intra-TAD ratio and similar number of bins. The blue and red lines connect median and mean values of the different groups, respectively. (b) Violin plots of intra-TAD ratios of the highly expressed bins and lowly expressed bins, defined as the genomic bins containing TSSs of genes whose FPKM values rank within top and bottom 1,000, respectively. (c) Distribution of Spearman correlations between gene expression and intra-TAD ratio of genomic bins in individual TADs. Black dotted curve shows the fitted normal distribution. The vertical black dotted line shows the mean of Spearman correlations. The vertical red dotted line shows zero Spearman correlation. (d) Violin plots of gene expression levels ($\log_2$(FPKM+0.01)) in domain surface bins and domain core bins. p-values in all panels are calculated using the two-sided Mann-Whitney U test.

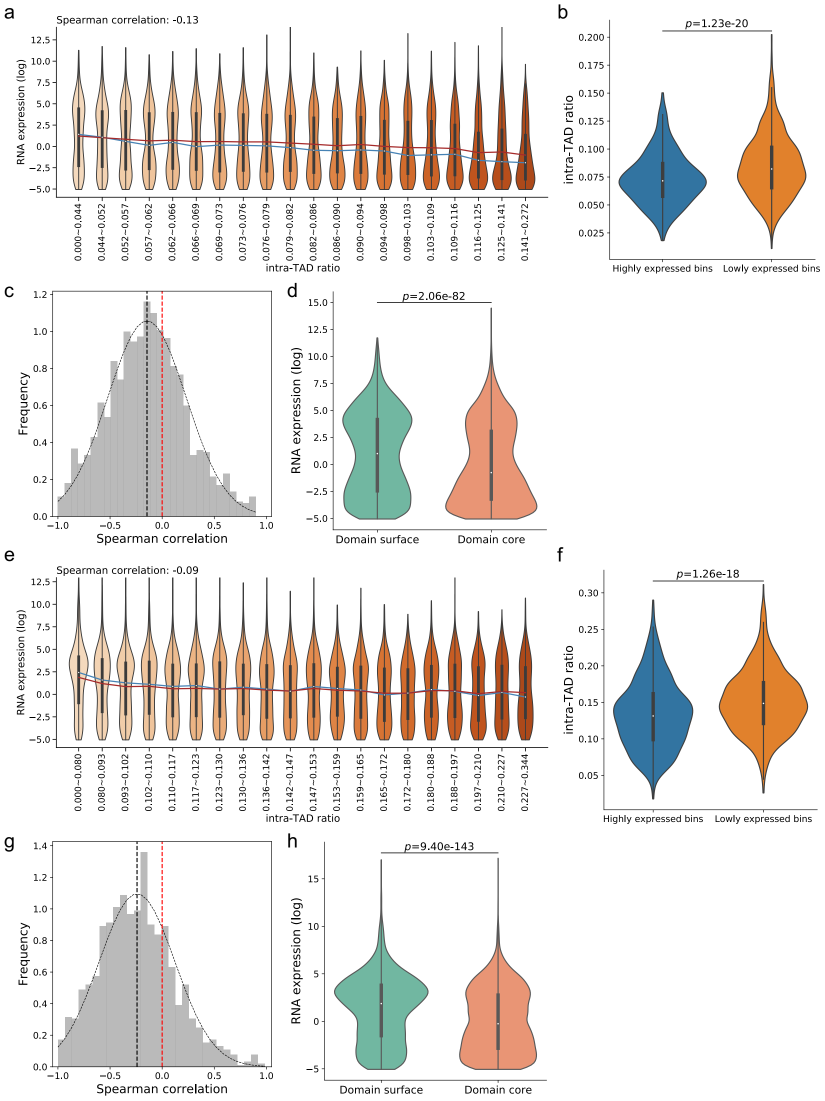

Figure S23: Intra-TAD ratio and gene expression have an inverse relationship in GM12878 (a-d) and mESC (e-h) even when genes with extremely low expression (RPKM<0.02) are ignored. (a,e) Violin plots of gene expression ($\log_2$(FPKM+0.01)) of TSS bins in groups with increasing intra-TAD ratio and similar number of bins. The blue and red lines connect median and mean values of the different groups, respectively. (b,f) Violin plots of intra-TAD ratios of the highly expressed bins and lowly expressed bins, defined as the genomic bins containing TSSs of genes whose FPKM values rank within top and bottom 1,000 after TSS bins with extremely low expression (RPKM<0.02) are excluded, respectively. (c,g) Distribution of Spearman correlations between gene expression and intra-TAD ratio of genomic bins in individual TADs. Black dotted curve shows the fitted normal distribution. The vertical black dotted line shows the mean of Spearman correlations. The vertical red dotted line shows zero Spearman correlation. (d,h) Violin plots of gene expression levels ($\log_2$(FPKM+0.01)) in Domain surface bins and Domain core bins. p-values in all panels are calculated using the two-sided Mann-Whitney U test.

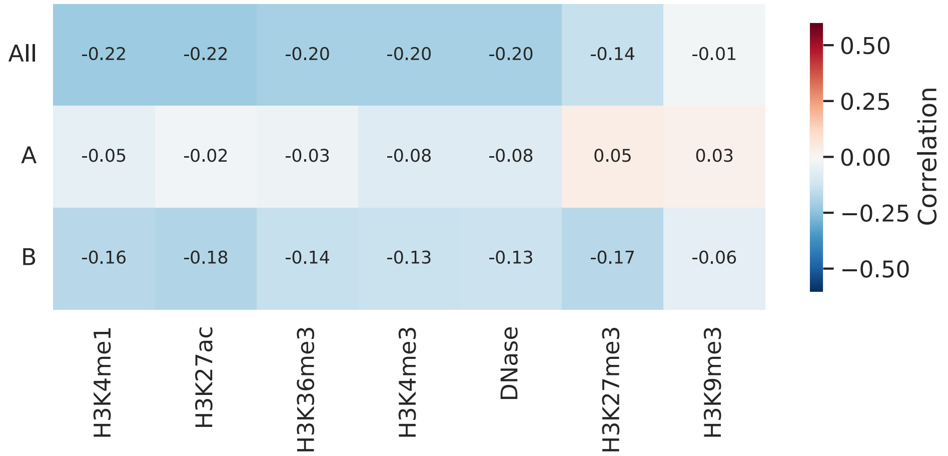

Figure S24: Spearman correlation between intra-TAD ratio and DNase-seq signals and 6 histone modifications across either all genomic bins or only bins in a genomic compartment in mESC. The columns are sorted by the correlation values when all TADs are considered (in the "All" row).

a

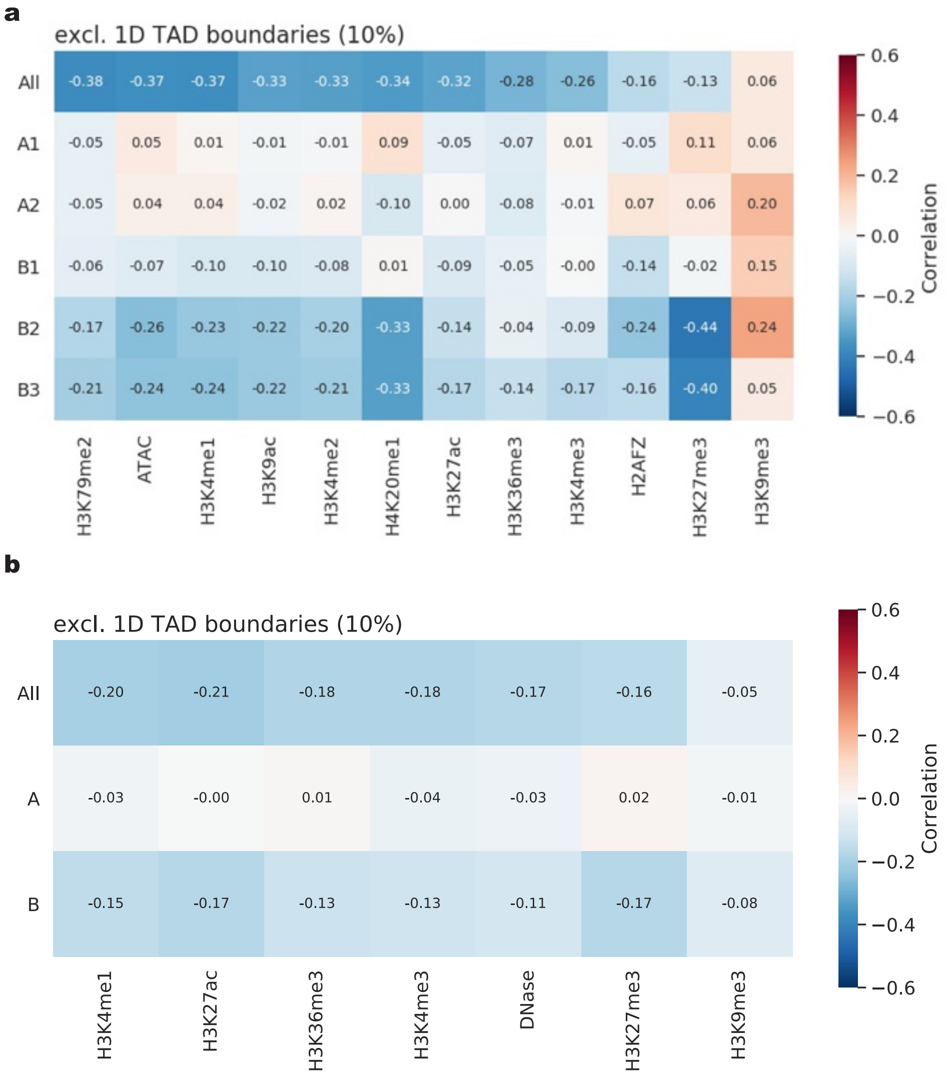

Figure S25: Spearman correlation between intra-TAD ratio and chromatin accessibility and histone modifications in GM12878 (a) and mESC (b) when 1D TAD boundaries are excluded. In each panel, the columns are sorted by the correlation values when all TADs are considered (in the "All" row).

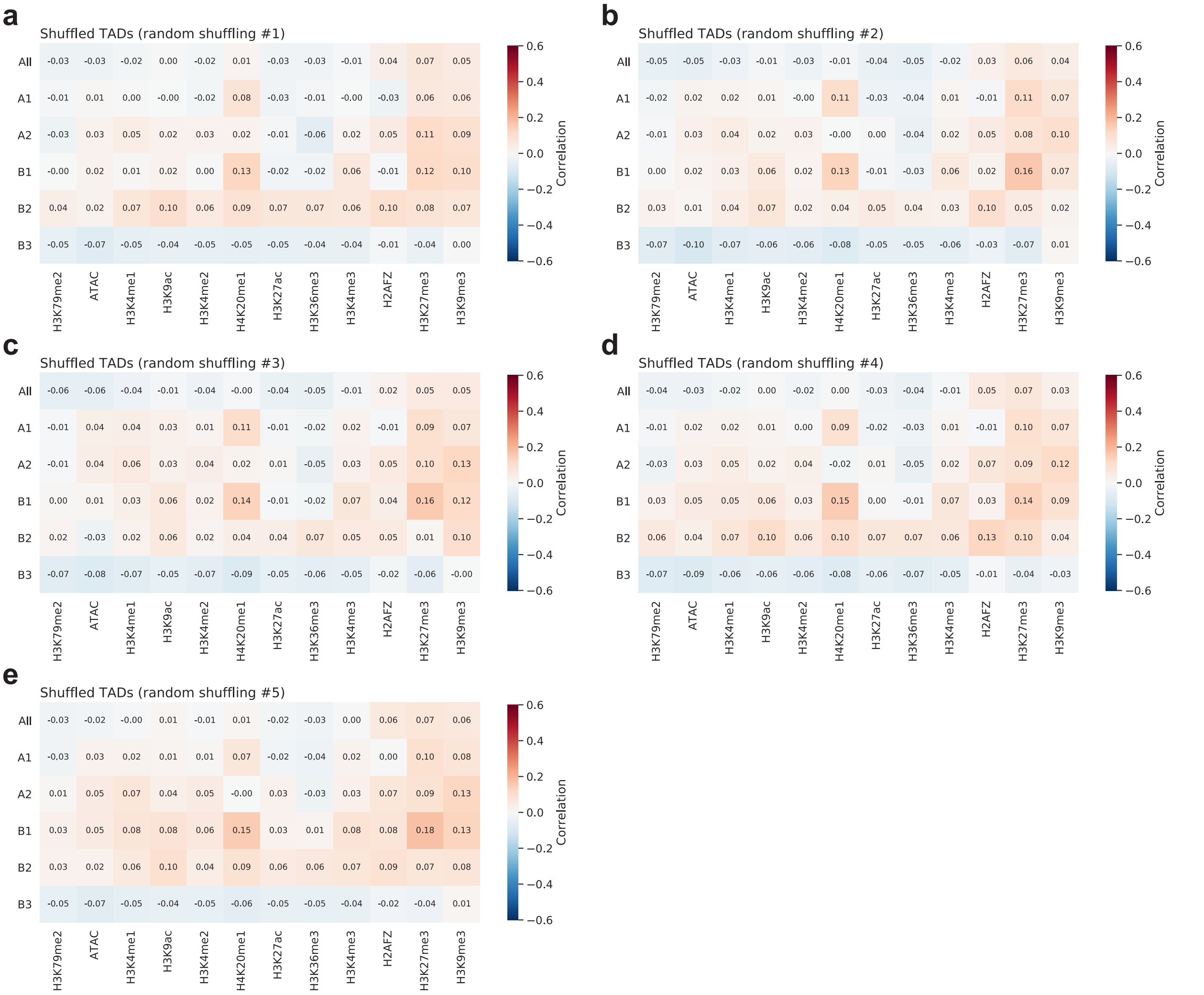

Figure S26: Spearman correlation between intra-TAD ratio and ATAC-seq signals and 11 histone modifications across either all genomic bins or only bins in a genomic subcompartment, with intra-TAD ratio being calculated using five sets of shuffled TADs. In each panel, the columns are sorted by the correlation values when all TADs are considered (in the "All" row).

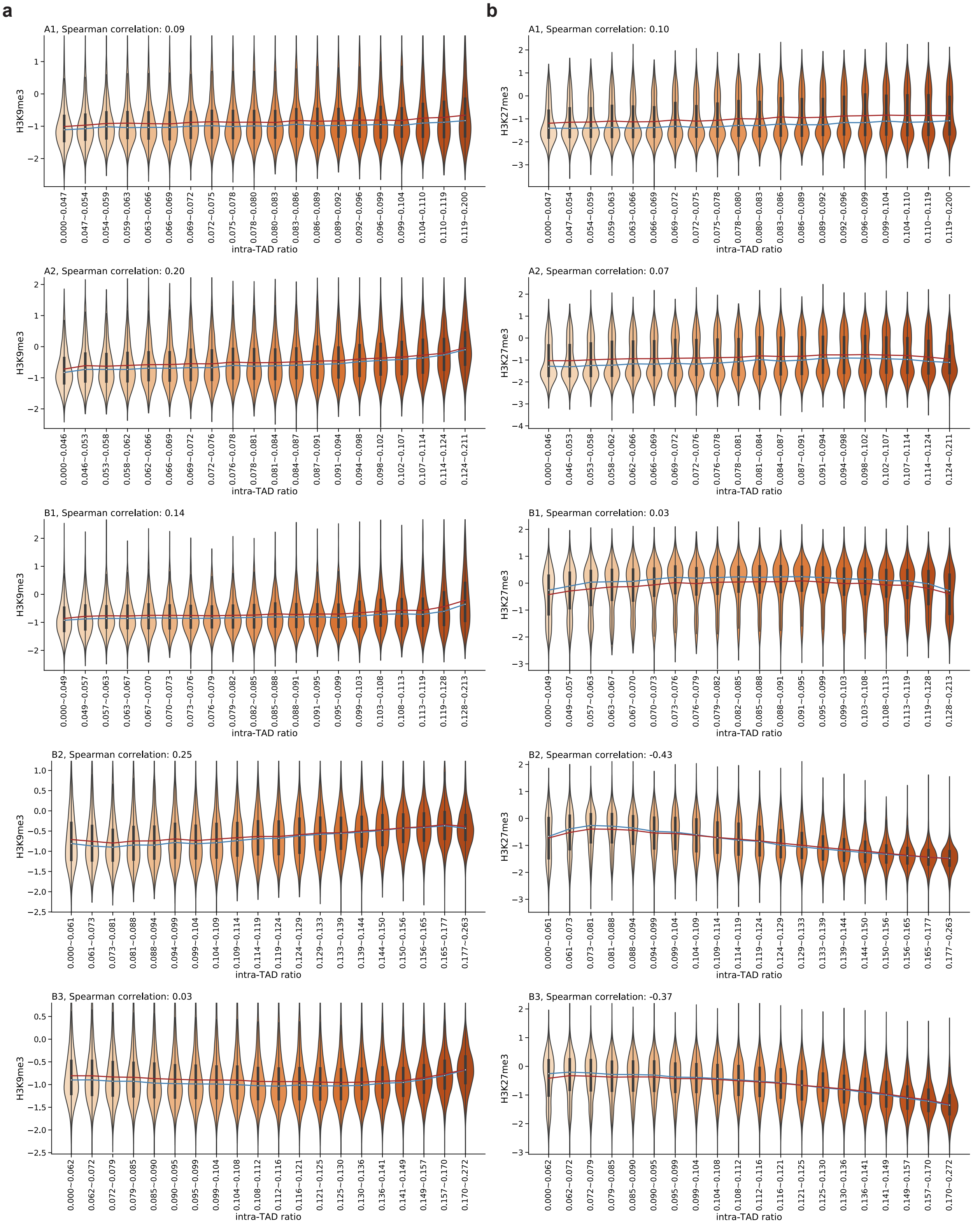

Figure S27: Violin plots of H3K9me3 (a) and H3K27me3 (b) signals in groups with increasing intra-TAD ratio and similar number of bins in individual subcompartments. y-axis shows $\log_2$(signal+0.01). The blue and red lines connect median and mean values of the different groups, respectively.

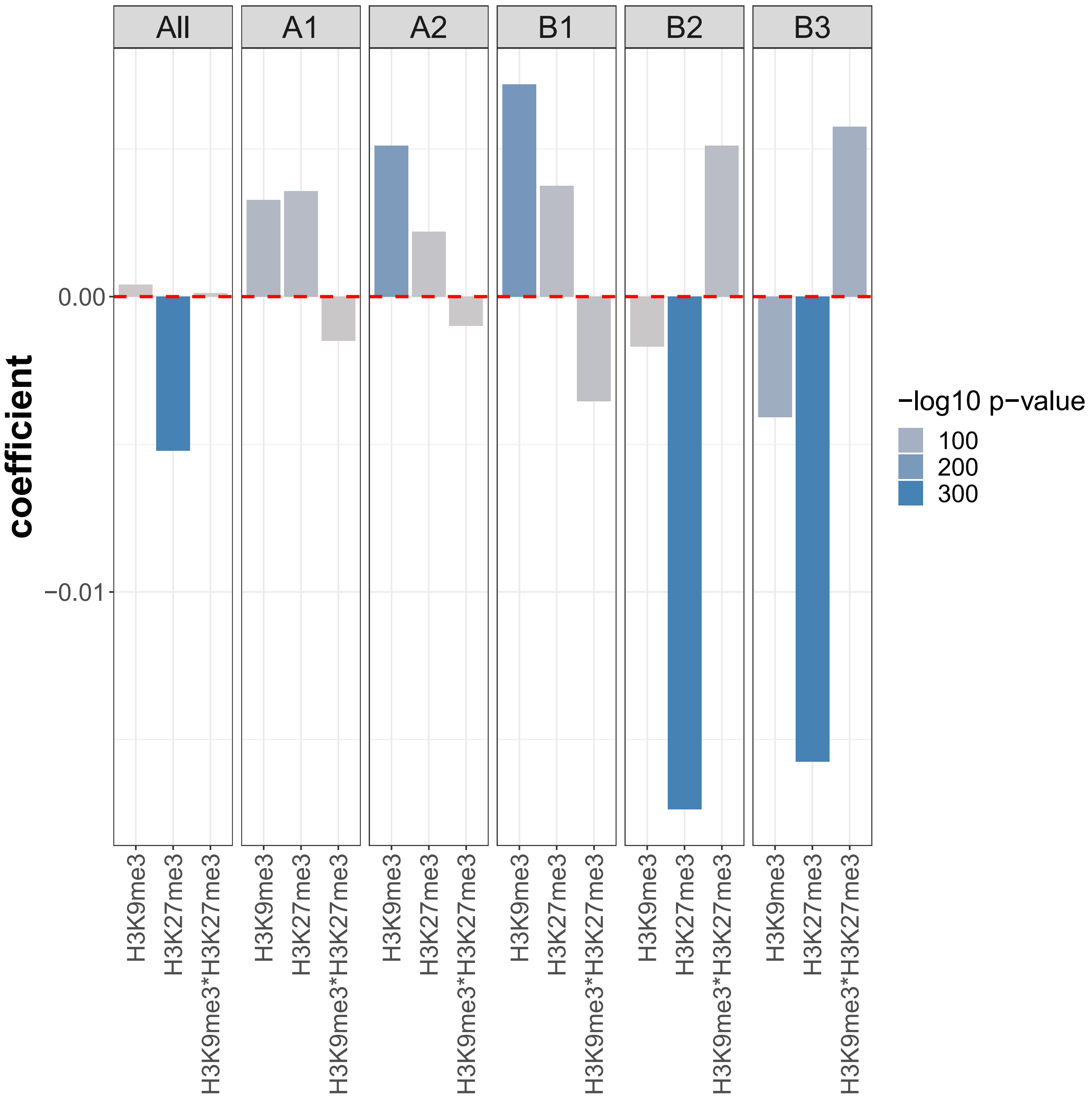

Figure S28: Coefficients of the linear regression models with H3K9me3, H3K27me3, and their interaction term as explanatory variables and intra-TAD ratio as the response variable, considering either all TADs or only TADs in a genomic subcompartment. The explanatory variables were all standardized individually before fitting the linear regression models.

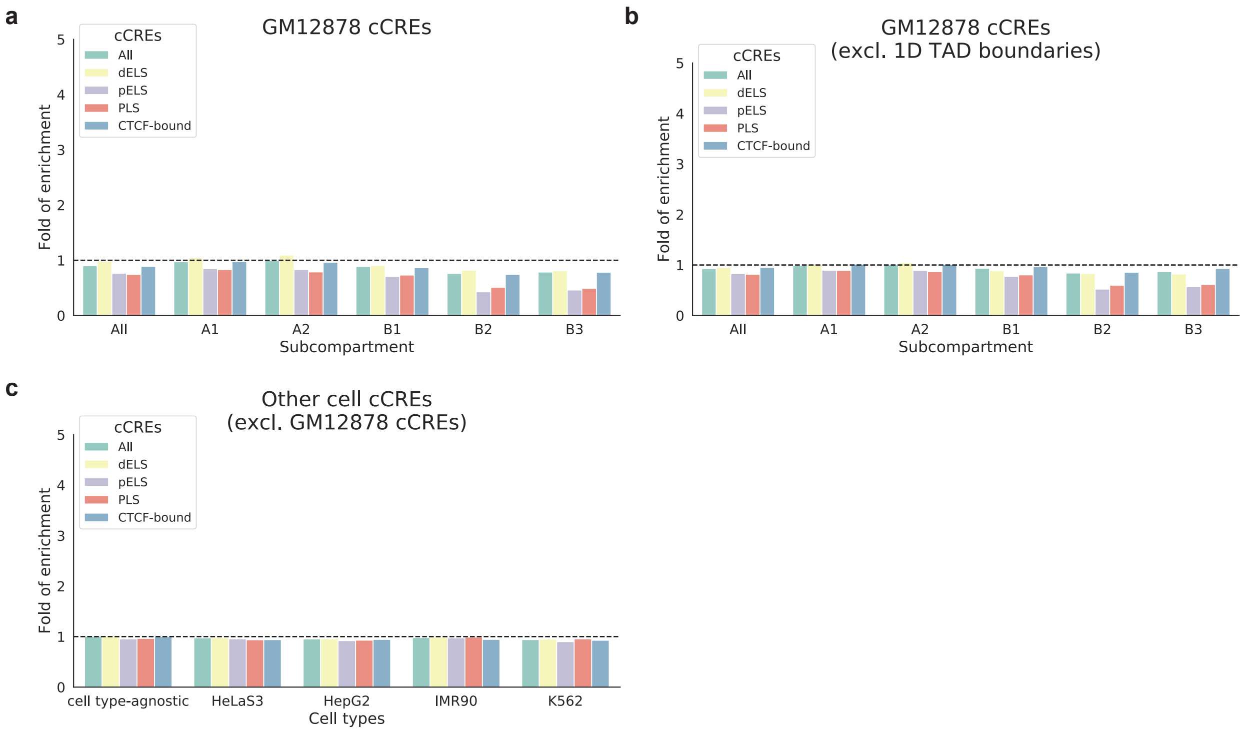

Figure S29: Depletion of CREs in chromatin domain cores. (a) Fold of enrichment of cCREs on domain cores in GM12878. (b) Fold of enrichment of cCREs on domain cores in GM12878 when the regions around TAD boundaries are excluded. (c) Fold of enrichment of cCREs on domain cores when the cCREs are cell type-agnostic or defined in other cell types and the GM12878-specific cCREs are excluded. cCREs All: all categories of cCREs; dELS: distal enhancer-like signatures; pELS: proximal enhancer-like signatures; PLS: promoter-like signatures; CTCF-bound: bound by CTCF.

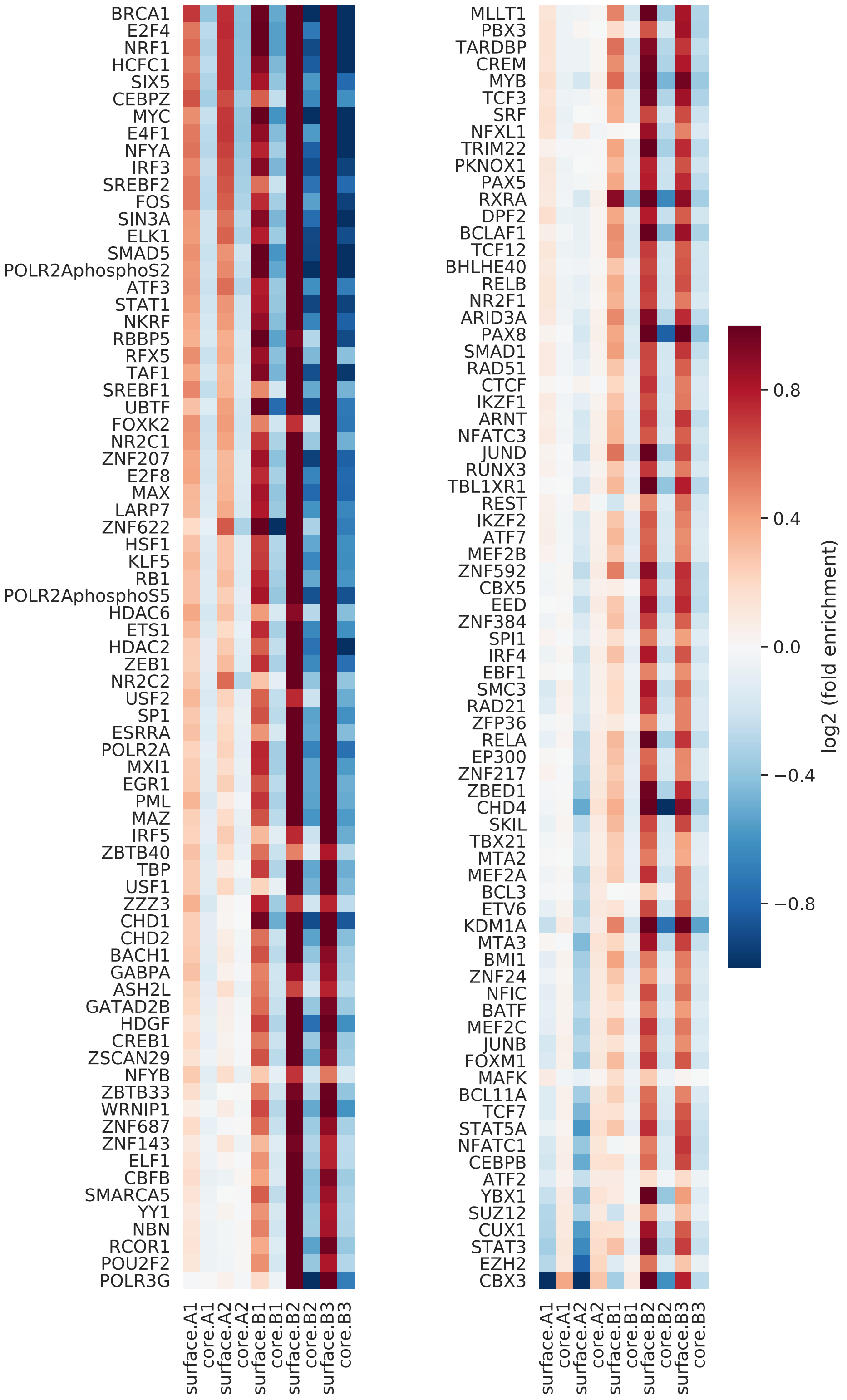

Figure S30: Heatmap showing log2(enrichment fold) of transcription factor binding sites in individual subcompartments on domain surfaces (odd-numbered columns) and domain cores (even-numbered columns) in GM12878.

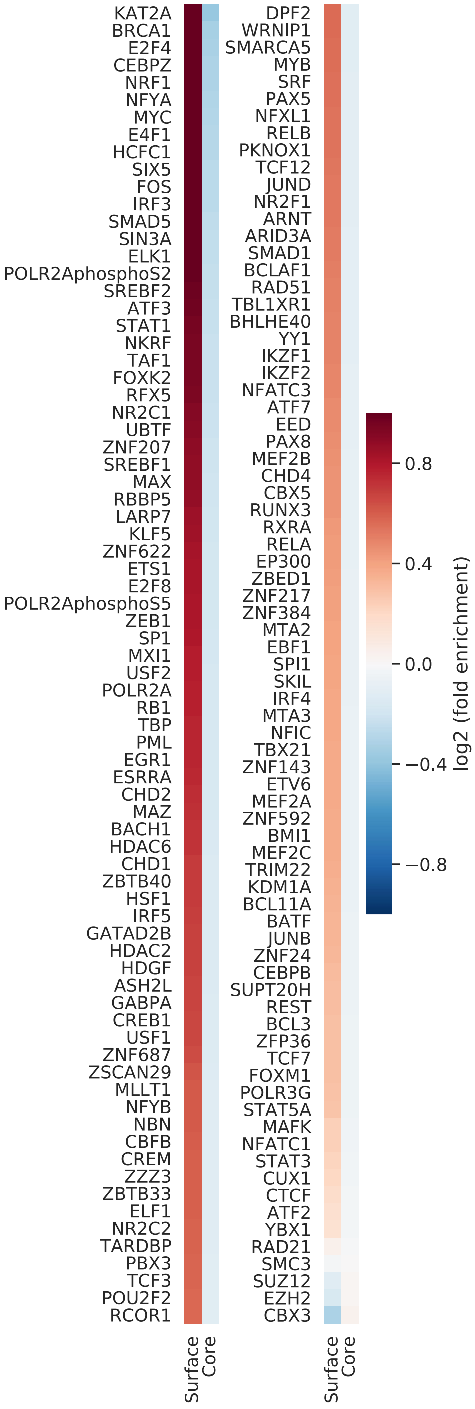

Figure S31: Heatmap showing log2(enrichment fold) of transcription factor binding sites on domain surfaces and domain cores in GM12878 when 1D TAD boundaries are excluded.

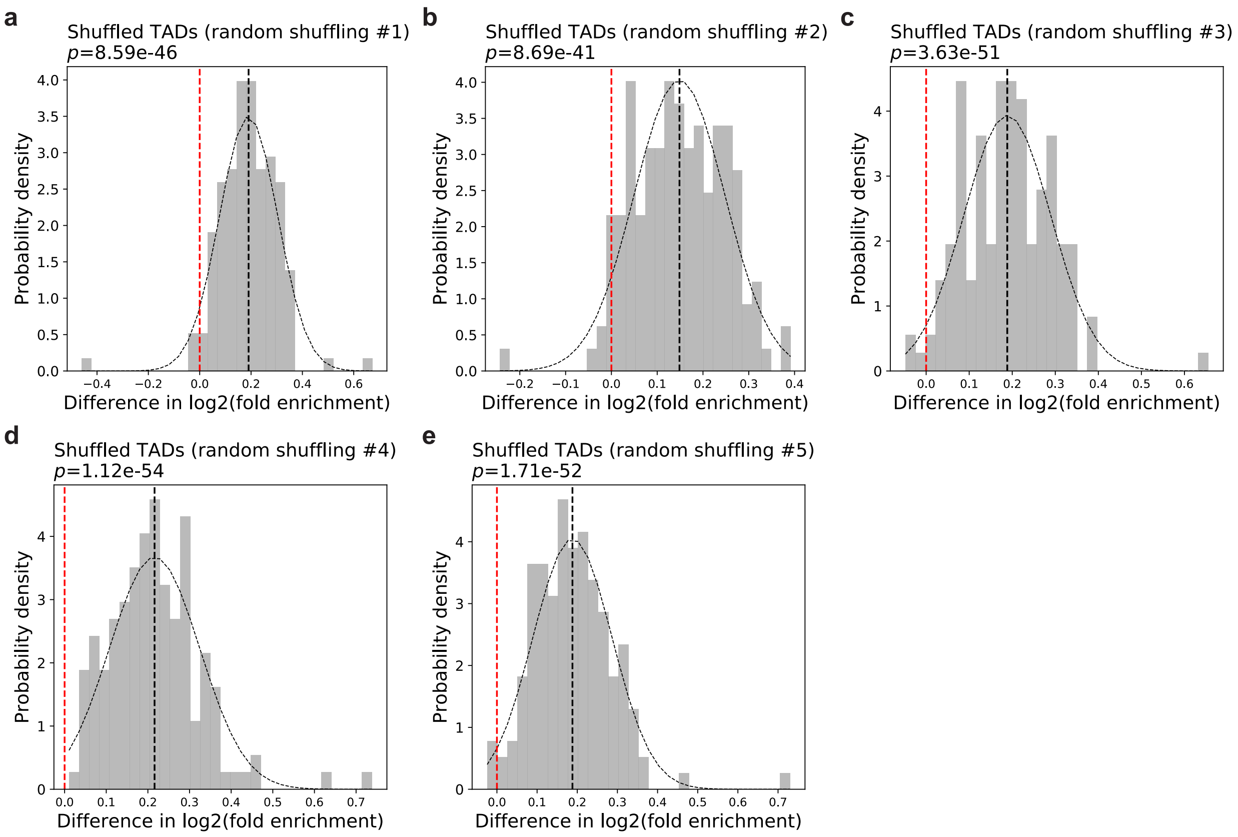

Figure S32: Distribution of difference in log enrichment fold of transcription binding sites on chromatin domain surfaces defined by intra-TAD ratio of real TADs and shuffled TADs. For each transcription factor, we calculated the log2 enrichment fold of transcription binding sites on domain surfaces defined by intra-TAD ratio of real TADs and shuffled TADs, respectively, and computed the difference (real TAD minus shuffled TAD). The distribution was plotted for all transcription factors. In all panels, black dotted curves show the fitted normal distribution. The vertical black dotted lines show the mean difference in log2(enrichment fold). The vertical red dotted lines show zero difference, as a reference. p-values are computed using the two-sided t-test.

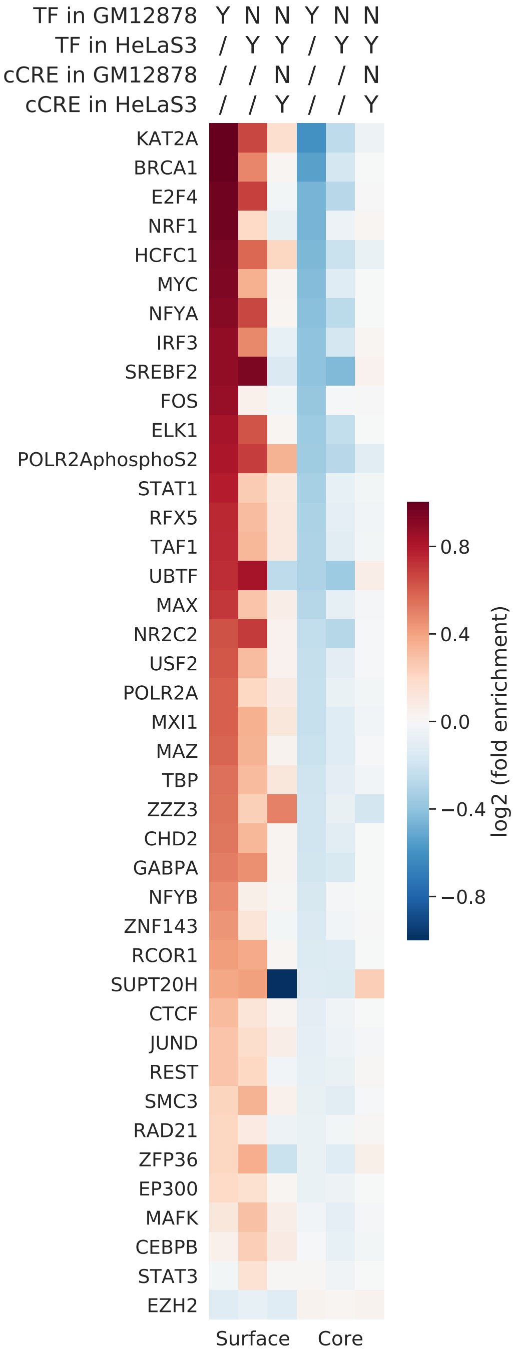

Figure S33: Heatmap comparing the locations of transcription factor binding sites and CREs in HeLaS3 and GM12878 relative to the TADs in GM12878. The first three columns show the enrichment of these locations on the GM12878 domain surfaces. The last three columns show the enrichment of these locations in the GM12878 domain cores. In the header, “Y” means only regions satisfying the condition are included, “N” means only regions not satisfying the condition are included, and “/” means the condition is not involved in defining the regions and thus no regions are excluded. The final set of regions is the intersection of the regions satisfying the different specified conditions.

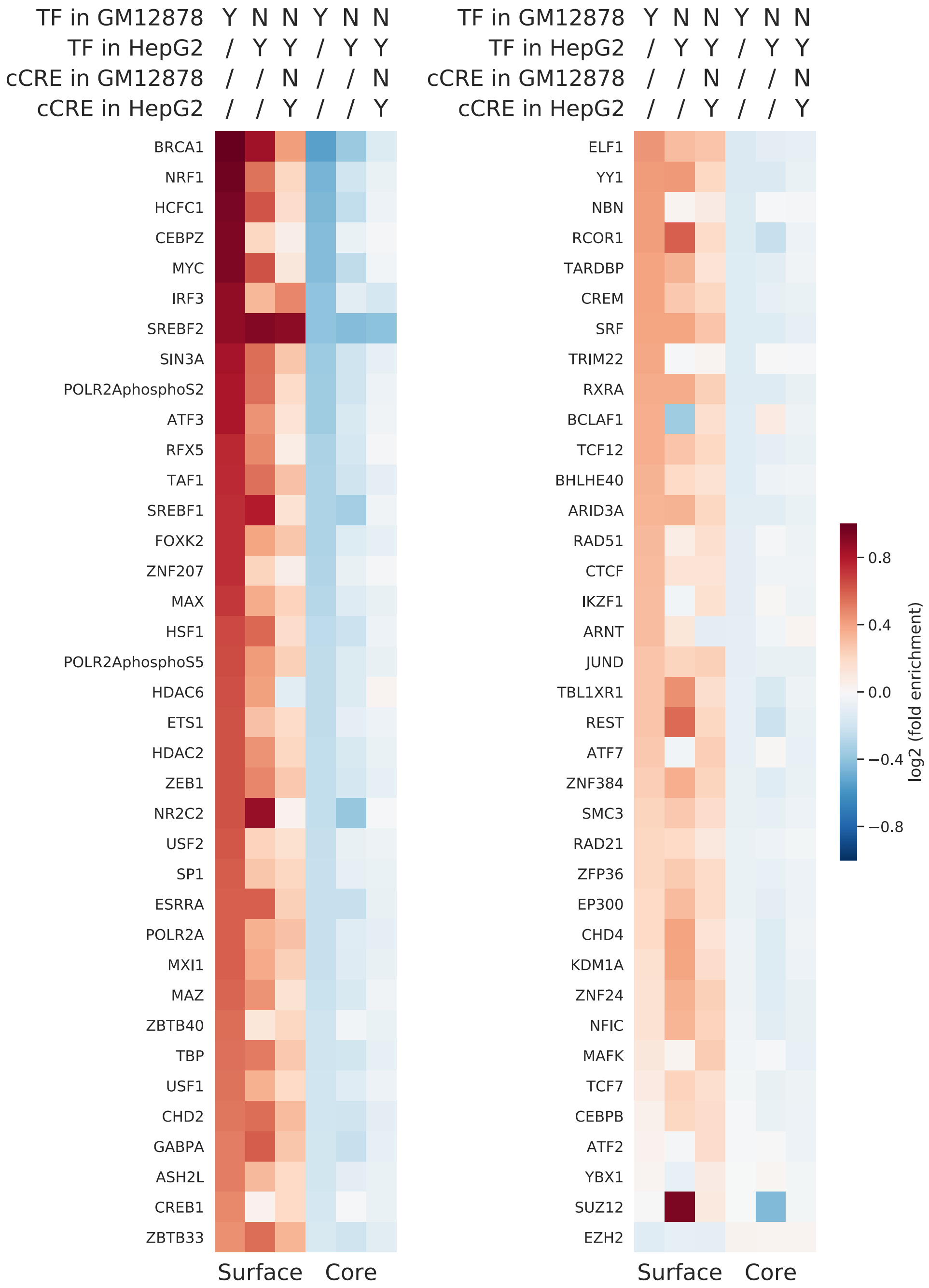

Figure S34: Heatmap comparing the locations of transcription factor binding sites and CREs in HepG2 and GM12878 relative to the TADs in GM12878. The first three columns show the enrichment of these locations on the GM12878 domain surfaces. The last three columns show the enrichment of these locations in the GM12878 domain cores. In the header, "Y" means only regions satisfying the condition are included, "N" means only regions not satisfying the condition are included, and "/" means the condition is not involved in defining the regions and thus no regions are excluded. The final set of regions is the intersection of the regions satisfying the different specified conditions.

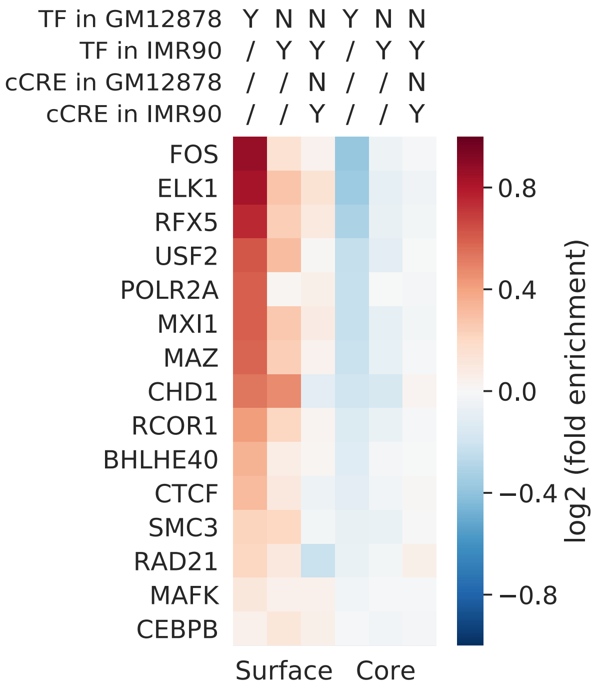

Figure S35: Heatmap comparing the locations of transcription factor binding sites and CREs in IMR90 and GM12878 relative to the TADs in GM12878. The first three columns show the enrichment of these locations on the GM12878 domain surfaces. The last three columns show the enrichment of these locations in the GM12878 domain cores. In the header, "Y" means only regions satisfying the condition are included, "N" means only regions not satisfying the condition are included, and "/" means the condition is not involved in defining the regions and thus no regions are excluded. The final set of regions is the intersection of the regions satisfying the different specified conditions.

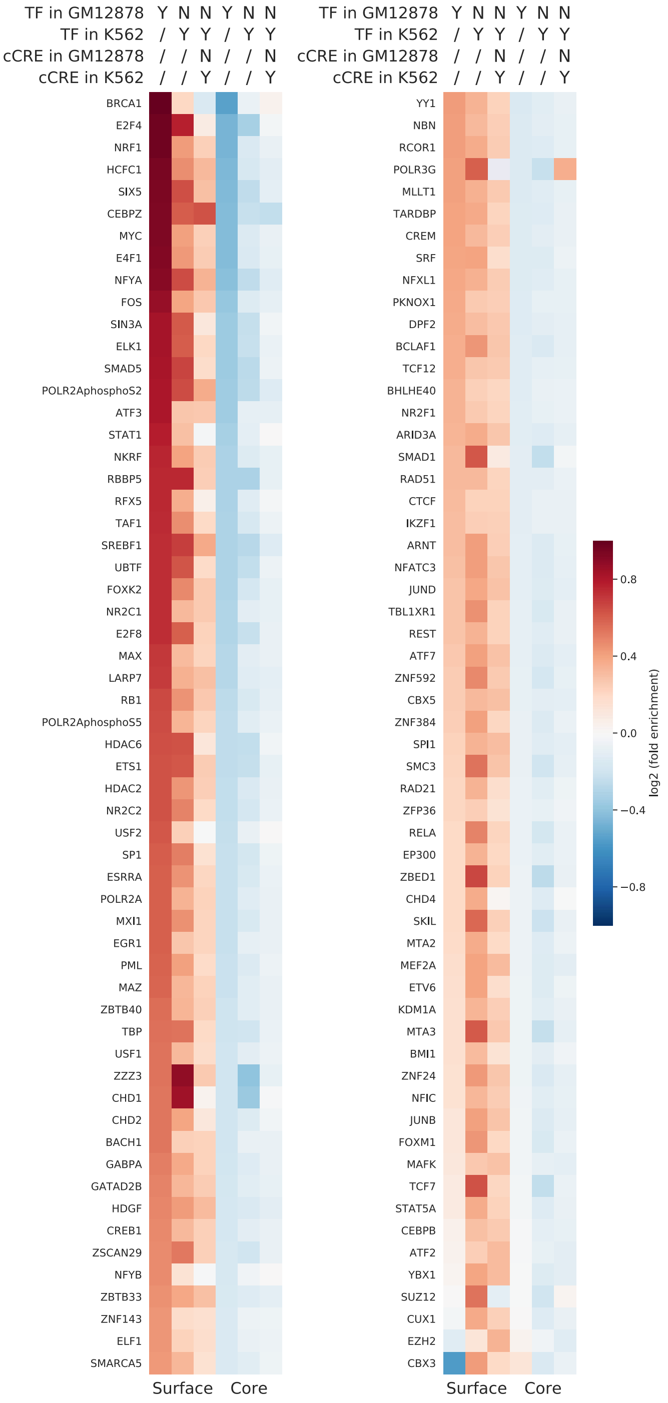

Figure S36: Heatmap comparing the locations of transcription factor binding sites and CREs in K562 and GM12878 relative to the TADs in GM12878. The first three columns show the enrichment of these locations on the GM12878 domain surfaces. The last three columns show the enrichment of these locations in the GM12878 domain cores. In the header, "Y" means only regions satisfying the condition are included, "N" means only regions not satisfying the condition are included, and "/" means the condition is not involved in defining the regions and thus no regions are excluded. The final set of regions is the intersection of the regions satisfying the different specified conditions.

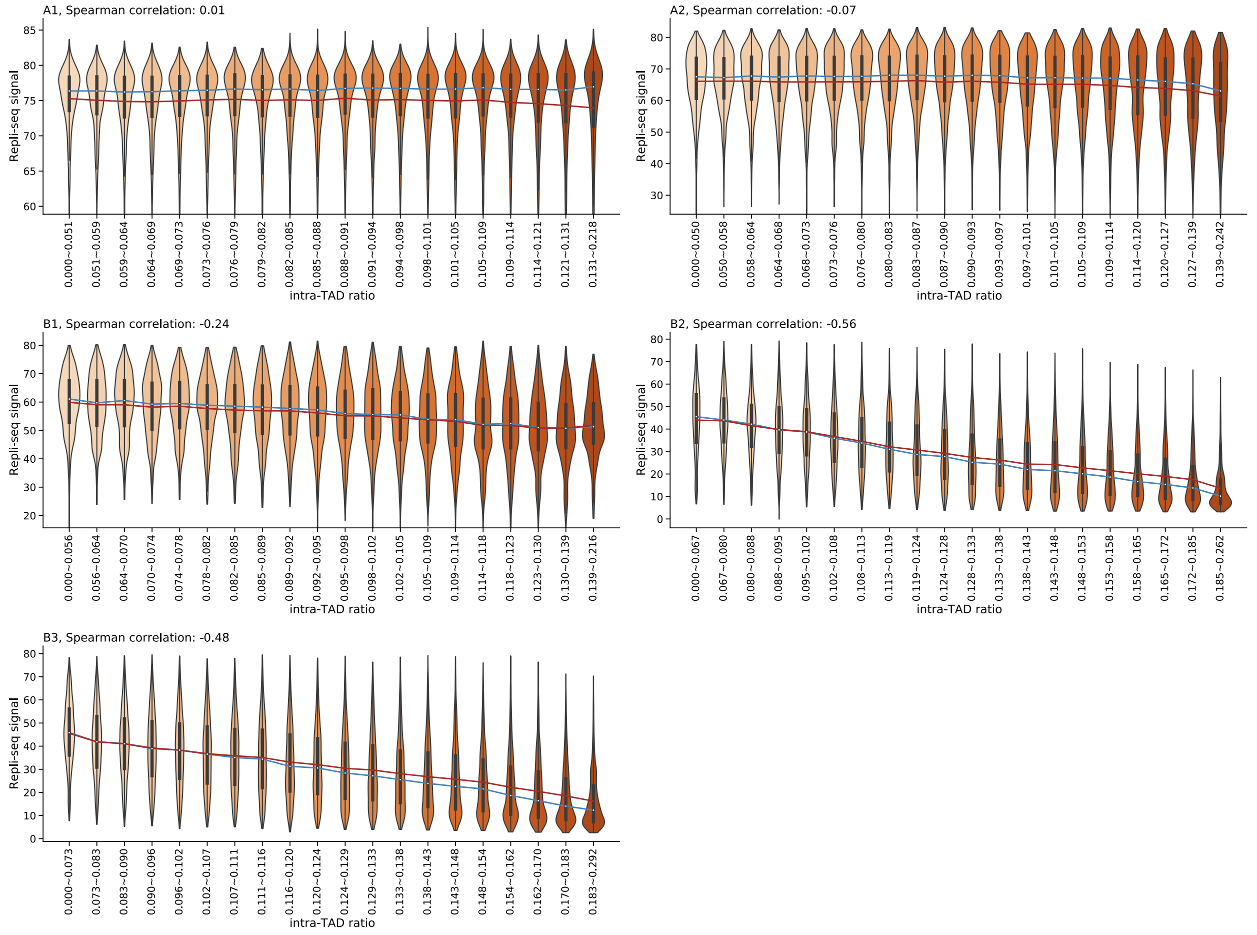

Figure S37: Violin plots of Repli-seq signals in groups with increasing intra-TAD ratio and similar number of bins in individual subcompartments. The blue and red line connects median and mean values, respectively, in each group.

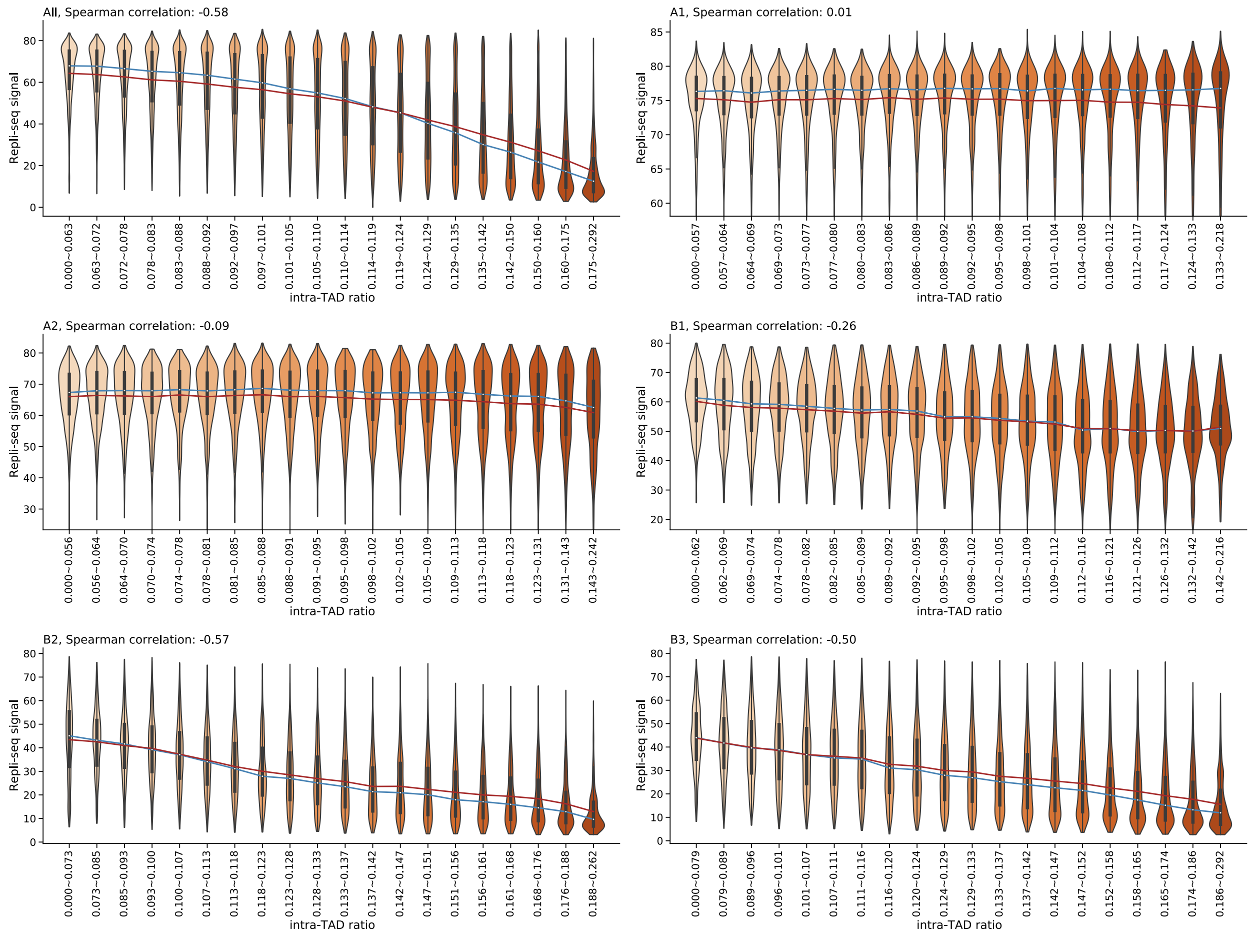

Figure S38: Violin plots of Repli-seq signals in groups with increasing intra-TAD ratio and similar number of bins when TAD boundaries are excluded. The blue and red line connects median and mean values, respectively, in each group.
